# Boron-containing helicenes as new generation of chiral materials: opportunities and challenges of leaving the flatland

**DOI:** 10.1039/d4sc01083c

**Published:** 2024-04-19

**Authors:** Agnieszka Nowak-Król, Patrick T. Geppert, Kenkera Rayappa Naveen

**Affiliations:** a Institut für Anorganische Chemie and Institute for Sustainable Chemistry & Catalysis with Boron, Universität Würzburg Am Hubland 97074 Würzburg Germany agnieszka.nowak-krol@uni-wuerzburg.de

## Abstract

Increased interest in chiral functional dyes has stimulated activity in the field of boron-containing helicenes over the past few years. Despite the fact that the introduction of boron endows π-conjugated scaffolds with attractive electronic and optical properties, boron helicenes have long remained underdeveloped compared to other helicenes containing main group elements. The main reason was the lack of reliable synthetic protocols to access these scaffolds. The construction of boron helicenes proceeds against steric strain, and thus the methods developed for planar systems have sometimes proven ineffective in their synthesis. Recent advances in the general boron chemistry and the synthesis of strained derivatives have opened the way to a wide variety of boron-containing helicenes. Although the number of helically chiral derivatives is still limited, these compounds are currently at the forefront of emissive materials for circularly-polarized organic light-emitting diodes (CP-OLEDs). Yet the design of good emitters is not a trivial task. In this perspective, we discuss a number of requirements that must be met to provide an excellent emissive material. These include chemical and configurational stability, emission quantum yields, luminescence dissymmetry factors, and color purity. Understanding of these parameters and some structure–property relationships should aid in the rational design of superior boron helicenes. We also present the main achievements in their synthesis and point out niches in this area, *e.g.* stereoselective synthesis, necessary to accelerate the development of this fascinating class of compounds and to realize their potential in OLED devices and in other fields.

## Abstract 2

Recent advances in synthesis have opened the way to a variety of boron helicenes. We highlight the main achievements in the synthesis of these chiral compounds and discuss their photophysical properties and potential as functional materials.

## Graphical abstract

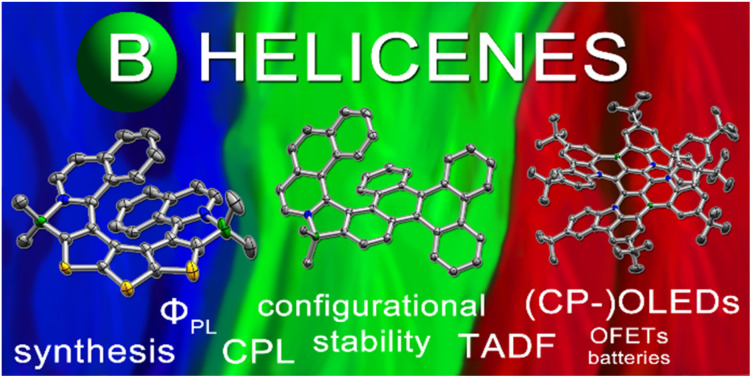

## 1. Introduction

Scientific interest in helicenes, polycyclic aromatic hydrocarbons (PAHs) composed of angularly fused aromatic or heteroaromatic rings, is growing exponentially,^1–7^ motivated in particular by their potential in chiral electronics.^8^ According to Web of Science, the number of reports on helicenes has nearly tripled within the last decade. Their inherent chirality, resulting from their screw-shaped structures gives rise to chiroptical properties, such as optical rotatory dispersion (ORD), circular dichroism (CD) and circularly polarized luminescence (CPL).^9,10^ These features, together with their electronic and charge–carrier properties make them interesting for a wide range of applications, *e.g.* in circularly polarized organic light-emitting diodes (CP-OLEDs),^11^ field-effect transistors (OFETs),^12^ spintronics,^13,14^ and as chiral switches.^15–17^ In addition, their structural features are of great utility in asymmetric catalysis,^18,19^ supramolecular chemistry,^20^ and molecular recognition.^21,22^

Approaches to promote and tune helicene properties include the helical or lateral extension of π-conjugated systems, attachment of electron-donating or -withdrawing substituents to their helical frameworks, or introduction of multiplicity by combining two or more helical scaffolds in a single molecule,^1,23–25^ leading to enhanced non-planarity, and improved intermolecular interactions. Another prominent strategy to modulate the properties of these PAHs is doping with p-block elements (*e.g.* S, N, O, P, Si)^7^ or transition metals (*e.g.* Pd, Ir).^26,27^ In general, introducing electron-deficient boron into π-conjugated structures leads to significant perturbation of electron density, lowers the lowest unoccupied molecular orbitals (LUMOs), and endows the scaffolds with superb optical properties.^28–31^ Despite these attractive features, boron helicenes have long lagged far behind. The lack of suitable synthetic methods to access these scaffolds and the air and water stability of some boron-containing PAHs can be pointed out as the main reasons.

The concepts of so-called structural constraint and kinetic stabilization with pivotal contributions from Yamaguchi,^32,33^ Marder,^34–36^ and other groups,^37–42^ have significantly influenced the development of stable boron-doped PAHs. The inherently reactive boron atom, when embedded in the inner part of the PAH, is stabilized by structural constraint. According to this principle, the formation of a tetracoordinated adduct with a nucleophile, *e.g.* water, becomes disfavored due to the unfavorable reorientation from a trigonal planar to a tetrahedral geometry of the PAH upon coordination of a nucleophile. On the other hand, the boron atom placed on the edge of a helical scaffold is often stabilized by kinetic shielding provided by a bulky aryl group.^40,43^ The simultaneous introduction of N or O atoms as B–N and B–O units, decreasing the Lewis acidity of boron, can effectively overcome the innate reactivity of three-coordinate organoboranes toward oxygen and moisture, providing PAHs with sufficient stability under ambient conditions. These strategies also proved useful in the synthesis of different boron-containing helicenes. In the examples presented in this article, the reader will identify all these stabilization methods.

**Fig. 1:**
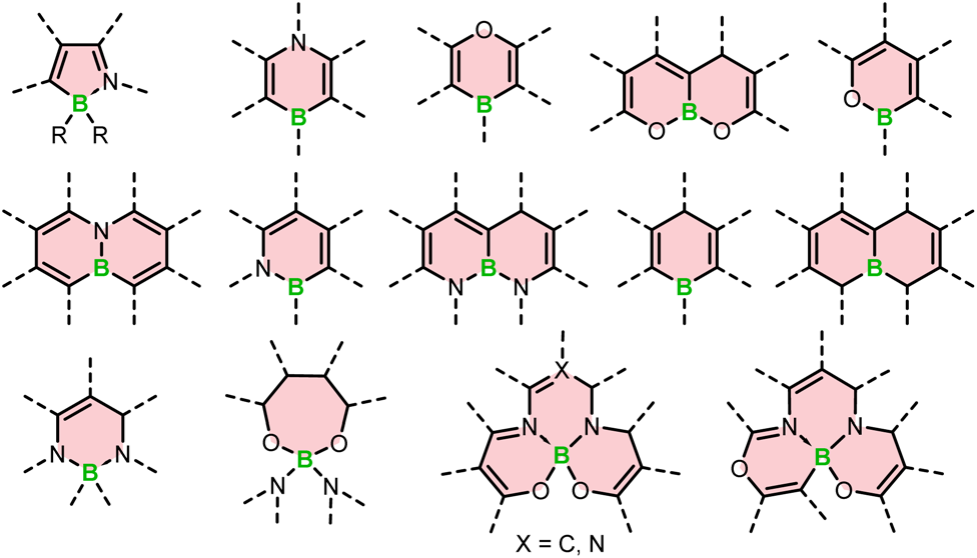
Types of boracycles embedded in boron-containing helicenes.

The search for outstanding chiral materials have sparked interest in boron helicenes. The progress in this field is truly remarkable. It has been driven not only by the development of synthetic methods dedicated to chiral derivatives, but also general and versatile protocols for the synthesis of boracycles. It is worth noting that a few years ago, there were only a handful of configurationally stable derivatives. Currently, we are witnessing the emergence of a variety of derivatives with rich and diverse structural features. These include azaboroles with four-coordinated boron and derivatives containing three-coordinated boron with B–O, O–B–O, N–B, and N–B–N motifs, in addition to a well-known class of BODIPY dyes and helicenes composed of only carbon and boron atoms (see Fig. 1).

We would like to point out that boron-doped PAHs are often confused with boron-containing helicenes. Here, we decided to limit the examples to PAHs containing boron in a helical framework. The examples where boron is placed on the periphery of the helicene arrangement also show interesting properties but are beyond the scope of this article. Another restriction we make is to limit the discussion to the helicenes built from at least five angularly fused rings. The only exceptions are non-planar derivatives consisting only of boron and carbon atoms in the helical frameworks. Only recently, the very first configurationally stable derivative belonging to this subclass of boron helicenes has been published.^44^

While leaving the “flatland” offers exciting opportunities, it also comes with a number of additional challenges. The introduction of chirality into a molecular structure, increases its complexity. There are a number of factors that must be considered when designing a new compound, which determine the success of a particular research endeavor, with synthetic accessibility being the most critical. Based on our experience, some methods that are efficient for planar scaffolds, fail to deliver strained congeners. In addition, such compounds need to fulfil numerous requirements in order to be used as functional materials depending on the application of choice. In practice, it is extremely difficult to meet all these requirements in a single compound or material.

In this article, we discuss the opportunities of using chiral boron-containing materials in selected applications, and the challenges involved in designing compounds with tailored properties for their application in OLEDs. We also present the available synthetic repertoire for the synthesis of boron helicenes, along with the limitations of particular methods. To highlight the main achievements in the field of boron helicenes and critical aspects in the design of state-of-the-art materials, we have structured the article into sections focusing on general synthetic strategies for each subclass of boron helicenes, their relevant properties (configurational stability, emission efficiency, luminescence dissymmetry factor, emission maxima and color purity), and applications in technology (OLEDs, OFETs, and batteries). Configurational stability is an important parameter that determines the applicability of a given compound as a chiral material and should always be considered in the design of any functional chiral material. The other parameters are particularly important for their application in OLED devices. In addition to the two key parameters that determine the efficiency of a given emitter, *i.e.* the photoluminescence quantum yield (*Φ*_PL_) and the luminescence dissymmetry factor (*g*_lum_), we address the emission maximum, which defines the color of the emitted light and the color purity related to the width of the emission band.

We present a wide variety of boron-containing helically chiral compounds, though we do not aim to be exhaustive in our analysis. The selected examples are intended to portray the current state-of-the art in B-helicene emitters, explain the approaches that can be used to tune certain parameters and show the importance of these compounds for applications in future technologies.

We hope that this article will enhance the conceptual understanding of the application-oriented design of configurationally stable boron helicenes, provide guidance for the synthesis of superior boron-containing chiral functional materials, and stimulate research on these fascinating compounds.

## 2. Synthesis

While boron-containing helicenes hold great promise for many applications, including organic electronics, they are still underdeveloped due to synthetic challenges. The methods that are effective for planar derivatives are sometimes not suitable for synthesizing strained cores. In addition, the preparation of the required precursors, often sterically congested, may be a bottleneck of the entire synthetic sequence. Therefore, efficient methods to access these compounds are a prerequisite to advance the field of boron-containing chiral materials. In this section, we introduce the approaches and protocols that were adapted or developed to enable their synthesis. We grouped the B-doped helicenes into particular classes, for which the synthetic approaches are usually similar. These include helicenes containing five-membered azaborole rings, helicenes consisting of six-membered boron-containing rings, *i.e.* 1,4-oxaborinine, 1,2- and 1,4-azaborinines, helicenes with N–B–N and O–B–O motifs and chiral compounds composed of boracycles without any additional heteroatoms. Finally, we will discuss BODIPY dyes along with various strategies for achieving helical chirality in this compound class.

### 2.1. Azaborole helicenes

**Scheme 1:**
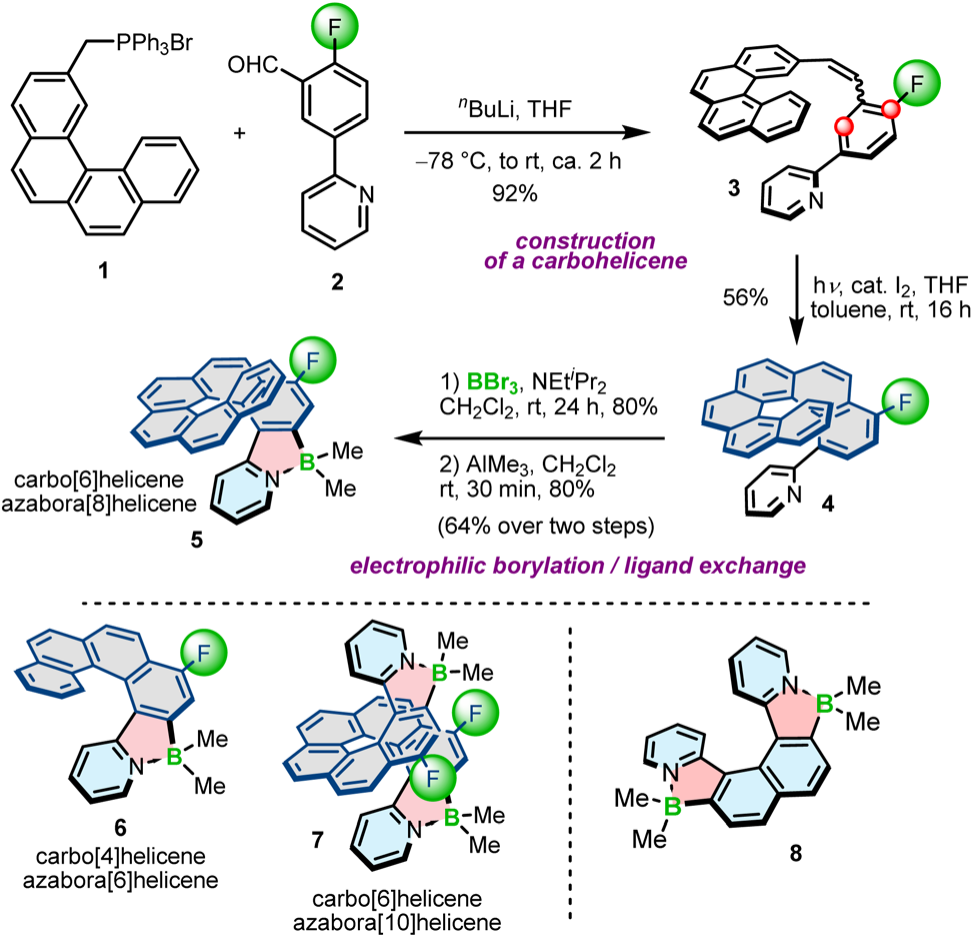
The synthetic route to azaborole helicenes through the extension of carbohelicenes. Azaborole rings are formed by electrophilic borylation with BBr_3_. Helicene 8 was synthesized from a dipyridyl-substituted naphthalene.

The potential of planar, achiral π-conjugated compounds containing five-membered 1,2-azaborole rings have been recognized in organic electronics, and photovoltaics.^31,45–47^ Azaboroles were also intensively studied by Wang and co-workers as photoswitches.^48–50^ To access these compounds, various methods have been developed depending on the type and electronic nature of constituent (hetero)aromatic rings and substituents on boron.^51–53^ In contrast to achiral derivatives, helically chiral azaborole helicenes have thus far been poorly investigated with only a limited number of synthetic approaches available to construct the azaborole ring. In principle, there are three general methods to synthesize azaborole helicenes. The most prominent approach involves nitrogen-directed electrophilic borylation under modified Murakami's conditions.^51^ According to this method, a 2-phenylpyridine derivative is reacted with BBr_3_ in the presence of a bulky tertiary amine, followed by the ligand exchange on boron. This method was used by Crassous and co-workers to prepare a set of azabora[*n*]helicenes consisting of an even number of angularly fused rings and bearing methyl substituents on boron.^54^ Compounds 5–7 bearing one (*n* = 6, 8) or two (*n* = 10) boron atoms were prepared by borylation of the corresponding carbo[4]- or [6]helicenes (colored in grey) appended with either a single or two pyridyl rings at the sterically hindered positions, followed by methylation with trimethylaluminium in excellent yields of 66–89% over two steps (Scheme 1). In this approach, reactions with BBr_3_ extended already existing helicene scaffolds. The required ligands (*e.g.*4) were prepared by the Witting reaction of (arylmethyl)triphenylphosphonium bromides (*e.g.*1) with 2-fluoro-5-(2-pyridinyl)benzaldehyde (2) and the subsequent photocyclization of the resulting stilbene derivatives (*e.g.*3). The introduction of the fluoride substituents into the precursors was inevitable to block one of two reactive sites (marked with red circles in Scheme 1) on the benzene ring and avoid the formation of regioisomeric products. In contrast to 5–7, the synthesis of azabora[6]helicene 8 did not require core substitution. The borylation of the corresponding dipyridyl-substituted naphthalene and the ligand exchange on boron afforded 8 in 55% yield. This compound is, however, prone to racemization (see Section 3.1).

**Scheme 2:**
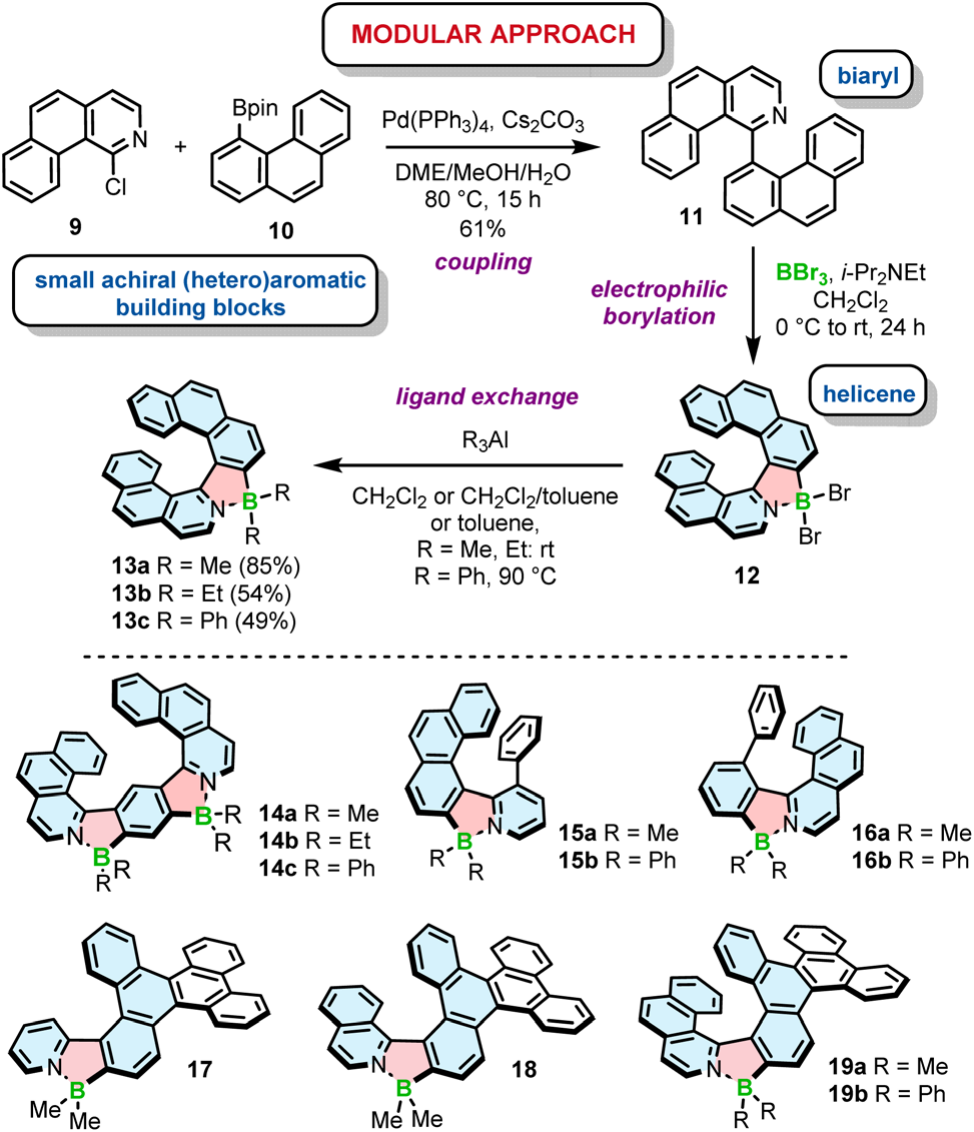
Modular approach to the synthesis of azaborole helicenes. Azaborole rings are formed by electrophilic borylation with BBr_3_.

Electrophilic borylation also lies at the heart of our modular approach (Scheme 2). In this general approach, which we proposed for the synthesis of azaborole helicenes, the key biaryl or oligoaryl intermediates (*e.g.*11) are assembled from small achiral building blocks (*e.g.*9 and 10). The formation of azaborole helicenes bearing halide substituents on boron (*e.g.*12) is executed by reacting these intermediates with a boron reagent at the late stage of the synthesis. While some of the precursors may be indeed axially chiral (*vide infra*), it is not until the boron atom is introduced that the helicene is constructed. We used this approach to synthesize a plethora of azaborole helicenes, such as single helicenes, multiples, laterally extended and truncated derivatives.^55–57^ The two-step reaction sequence, *i.e.* the borylation of the bi- or tertiary ligands with BBr_3_ in the presence of *N*,*N*-diisopropylethylamine (DIPEA) and the reaction with AlR_3_, afforded the target compounds bearing alkyl (methyl or ethyl) or phenyl substituents (*e.g.*13–19) in 34–91% yields. Here, we also tested diorganylzinc reagents, although they proved inferior to AlR_3_. The reaction of 12 with Et_2_Zn was more sluggish, had to be carried out at 70 °C and provided 13b in lower yields when compared to the analogous reaction with Et_3_Al. It is also important to note that the introduction of aryl substituents is more demanding than of alkyl groups. As opposed to Murakami's protocol, in which the ligand exchange with both alkyl and aryl substituents is carried out at room temperature, the introduction of Ph groups into the azaborole helicene required the temperature as high as 90 °C (Scheme 2). The difference in reactivity of BBr_2_ complexes toward both types of reagents is also reflected in the reaction times. While the substitution with Me can be shortened to 5 min, the corresponding reactions with Ph_3_Al are typically conducted for 1.5–4 h.

**Scheme 3:**
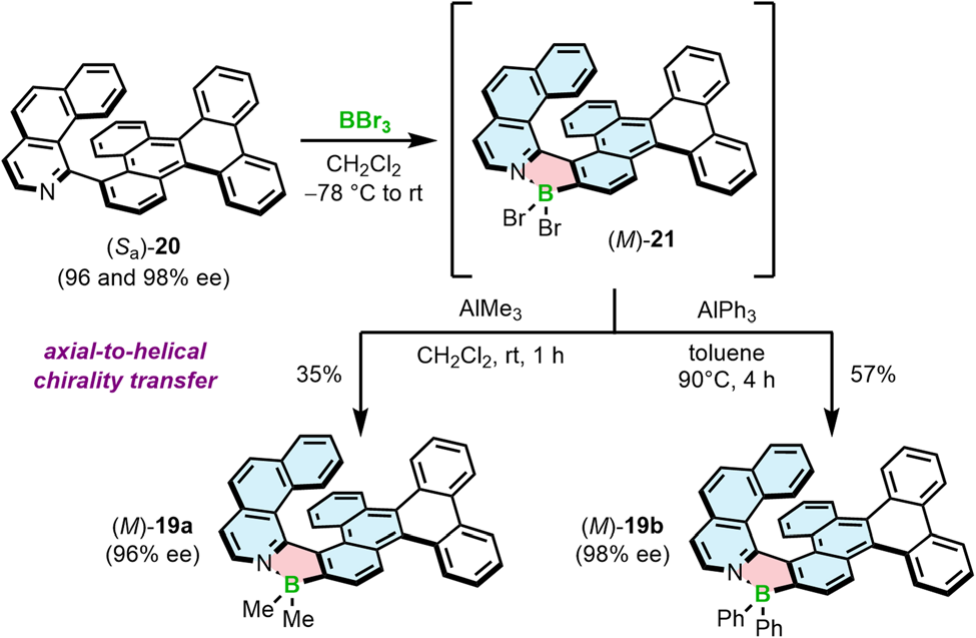
Stereospecific synthesis of azaborole helicenes by axial-to-helical chirality transfer for the selected enantiomer of biaryl 20.

We have also demonstrated that the synthesis of azaborole helicenes can be carried out in an enantiospecific fashion by axial-to-helical chirality transfer from enantioenriched axially chiral ligands (Scheme 3).^58^ Due to the enhanced rotational freedom of biaryls compared to the target helicenes, the rotation about the chiral axis may proceed in either sense leading to two plausible enantiomerization pathways. One of them is a puckered helicene-type transition state (TS), in which the steric hindrance is produced by N and 2-C atoms. In the second TS, the steric clash is built between the N-heterocyclic ring of one moiety and a ring of another moiety, distant to a chiral axis. The latter one, inaccessible for helicenes, is energetically more feasible and results in significantly lower enantiomerization barriers of biaryls compared to the corresponding helicenes. Accordingly, Δ*G*^‡^_en_ of 20, determined by dynamic HPLC, was only 23.3 kcal mol^−1^ at 298 K *versus* 33.1 kcal mol^−1^ at 443 K for 19a. This barrier, although not high, was sufficient to resolve the enantiomers and convert them into the target helicenes with full retention of chiral information (the enantiomeric excess of 20 and 19 were equal in each case). This also held true for the synthesis of Ph derivatives due to the fact that the first critical step, borylation with BBr_3_, was carried out at low temperature (−78 °C to rt), where 20 does not racemize. Once formed, the BBr_2_ complex benefits from the high configurational stability of the azaborole helicene and can be derivatized at elevated temperatures, required *e.g.* to introduce aryl substituents. On the other hand, a homologue of 20, shorter by one ring, is labile under ambient conditions with Δ*G*^‡^_en_ only slightly below the minimal barrier required to resolve the enantiomers (see Section 3.1).

**Scheme 4:**
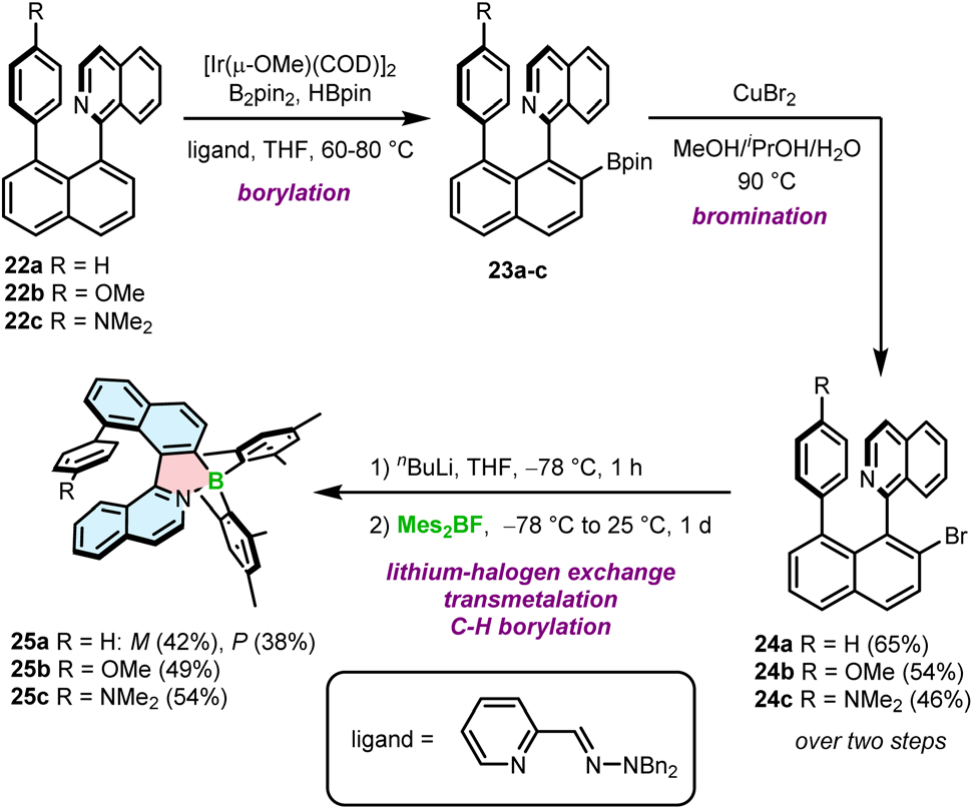
Synthetic route to azaborole helicenes with a ring attached at a sterically hindered position *via* Ir-catalyzed borylation.

The second approach is based on nitrogen-directed iridium-catalyzed borylation, followed by bromination under conditions developed by Hartwig,^59^ a subsequent halogen–lithium exchange and a reaction with a boron reagent (Scheme 4).^60^ This reaction sequence begins with the introduction of the Bpin group into 1-(naphthalen-1-yl)isoquinoline derivatives 22a–c. The reaction is carried out with B_2_pin_2_ in the presence of a catalytic amount of HBpin and [Ir(μ-OMe)(COD)]_2_ (COD = 1,5-cyclooctadiene) and the 2-pyridinecarboxaldehyde *N*,*N*-dibenzylhydrazone ligand at 60–80 °C.^61,62^ In the next step, Bpin-substituted compounds 23a–c are transformed into the corresponding bromides 24a–c in 46–65% yields using an aqueous solution of CuBr_2_ at 90 °C. These intermediates were then treated with *n*-BuLi at −78 °C, followed by the addition of Mes_2_BF providing target helicenes 25a–c in 38–54% yields. This approach is therefore suitable to synthesize helicenes bearing aryl substituents on boron, although the only group introduced so far using this method is mesityl (Mes). Interestingly, *rac*-24a could be resolved into its enantiomers and converted into the corresponding enantiomers of 25a with ee > 98%. However, these biaryls display low configurational stability resulting in partial racemization already at temperatures as low as −60 °C. Thus, it is more convenient to resolve target helicenes exhibiting higher Δ*G*^‡^_en_.

**Scheme 5:**
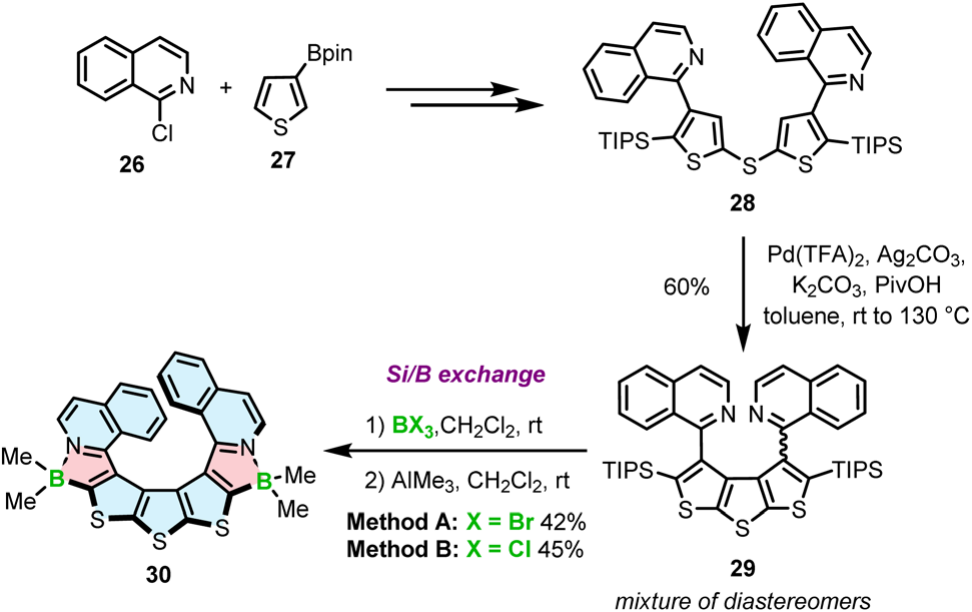
Synthesis of azaborathiahelicenes *via* silicon–boron exchange.

Recently, we reported a third approach for the synthesis of azaborole helicenes *via* silicon–boron exchange.^63^ This method was developed for the preparation of unprecedented azaborathia[9]helicene 30. The synthesis proceeded *via* highly congested atropisomeric teraryl 29, which was assembled from isoquinoline and thiophene building blocks 26 and 27 (Scheme 5). Due to the steric hindrance generated by four bulky substituents in close proximity, the fusion of the two outer rings to form the diethienothiophene (DTT) core was extremely challenging. Screening of various reactions and the optimization of reaction conditions allowed to identify the Pd-catalyzed dual C–H activation as an effective synthetic tool. Accordingly, the reaction using Pd(TFA)_2_, Ag_2_CO_3_ and K_2_CO_3_ in the presence of pivalic acid in toluene afforded triisopropylsilyl (TIPS)-substituted teraryl 29 in a highly satisfactory yield of 60%. The formation of the azaborole ring in the next step was performed between this key intermediate and BBr_3_ or BCl_3_*via* silicon–boron exchange. In contrast to poor-yielding electrophilic borylation (the yield of two steps including the reaction with BBr_3_ and the ligand exchange was below 5%), this direct method provided target helicene 30 in excellent yields of up to 45%. Importantly, this is an entirely new method for the preparation of azaboroles and the first demonstration of using TIPS in silicon–boron exchange reactions.

While this article focuses mainly on the last steps of the helicene syntheses, it is worth noting that the synthesis of azaborole helicenes is often hindered by the demanding preparation of the suitable sterically congested precursors. Therefore, the development of this compound class would benefit from new and efficient methods for the synthesis of atropisomeric intermediates, π-conjugated scaffolds with functional groups at demanding positions (bay region), and cross-coupling reactions between N-heterocycles and carbocyclic partners. The fact that α-borylated pyridines are typically unstable and exhibit poor reactivity in Suzuki coupling^64^ predetermines the positioning of certain functional groups in the building blocks (*e.g.* halogenated N-heterocycles and borylated carbocycle for Suzuki coupling) and often limits the available synthetic repertoire.

### 2.2. 1,4-Oxaborinine and 1,4-azaborinine helicenes

The relative orientation of boron and oxygen or nitrogen atoms and the type of bonds being formed defines the suitable synthetic method.

**Scheme 6:**
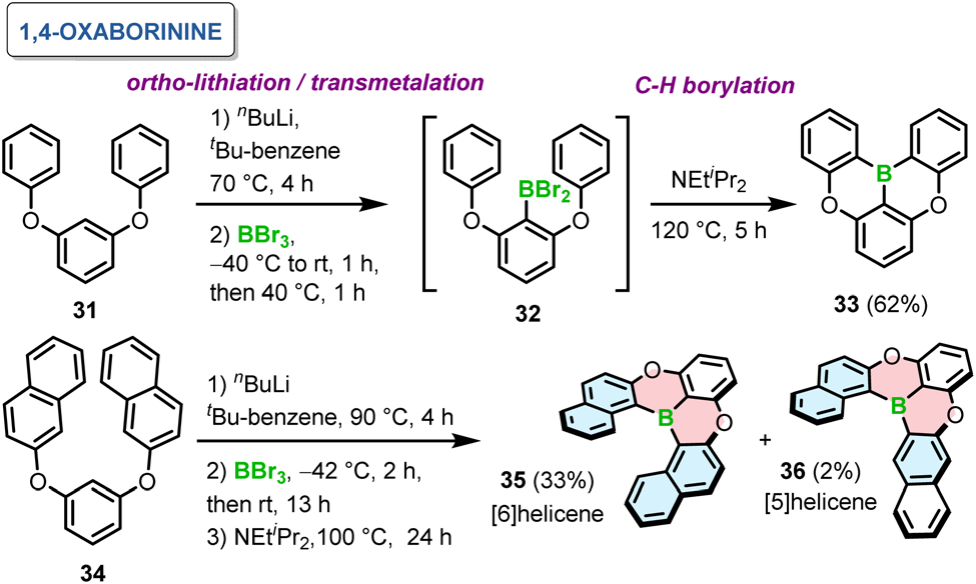
Synthesis of 1,4-oxaborinine-containing helicenes.

In 2015, Hatakeyama introduced a one-pot borylation of 1,3-diaryloxybenzenes to generate PAHs containing 1,4-oxaborinine rings.^65^ Directed *ortho*-lithiation of 31 using *n*-BuLi and transmetalation with BBr_3_ afforded 32. This species in the presence of Hunig's base led to double intramolecular C–H borylation at 120 °C to give 33 in 62% yield (Scheme 6). This compound, later abbreviated as DOBNA, constitutes nowadays a common motif of intensively studied narrowband emitters. The presence of a base and its type are crucial for the last step of this reaction sequence. For example, the yields in the absence of a base or in the presence of trimethylamine were low (13–16%), while they were comparable for 1,2,2,6,6-pentamethylpiperidine and *N*,*N*-dimethyl-*p*-toluidine. In general, the reaction is favored by weakly nucleophilic bases. It is noteworthy that this reaction proceeded without any Lewis acid activator, such as aluminum trichloride (AlCl_3_), presumably due to the presence of oxygen atoms, which enhance the nucleophilicity of the benzene rings. While such four-ring compounds derived from DOBNA exhibit highly attractive optical properties, their field of application is intrinsically limited to use as achiral emitters due to their low configurational stability. Notably, the method also proved effective in the synthesis of helicene 35 consisting of six angularly fused rings. The last step was carried out at somewhat lower temperature to afford 35 in 33%, in addition to tiny amounts of formally [5]helicene 36 (Scheme 6). The predominant ring closure at the more nucleophilic naphthyl 1-position of 34 indicates the preference for irreversible borylation under kinetic control.

**Scheme 7:**
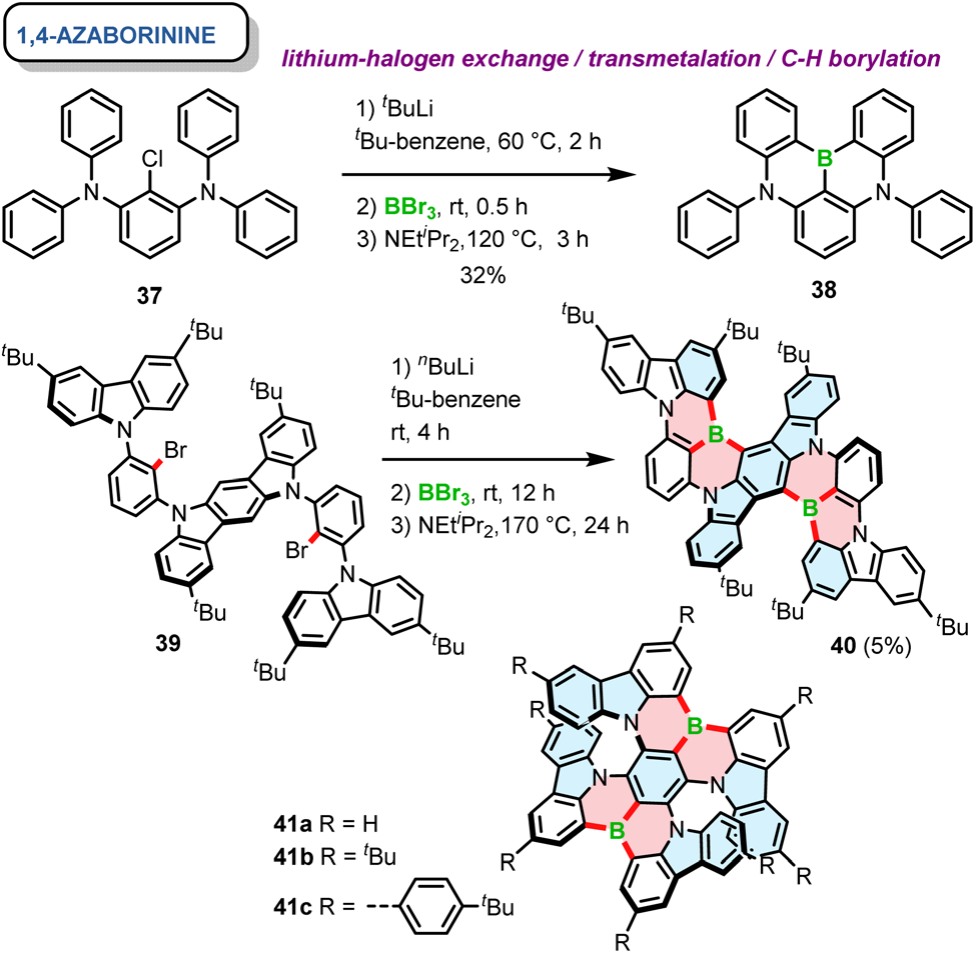
Synthesis of 1,4-azaborinine-containing helicenes. The bonds formed *via* borylative cyclization are colored in red.

Soon afterwards, the same group reported a core similar to DOBNA with nitrogen moieties replacing oxygen atoms. 38, referred to as DABNA-1, possesses one boron and two nitrogen atoms that form two 1,4-azaborinine rings combining the neighbouring phenyl groups.^66^ The one-pot synthesis of this achiral PAH was carried out using lithium–chloride exchange with *t*-BuLi, followed by electrophilic trapping with BBr_3_ and a double electrophilic C–H borylation in the presence of DIPEA. Since DABNA-1 showed improved thermally-activated delayed fluorescence (TADF) characteristics compared to DOBNA due to the enhanced multi-resonance (MR) effect (see Section 3.2), this motif was incorporated in a plethora of π-extended rigid systems, but only a handful of them are configurationally stable. The general synthesis of 1,4-azaborinine helicenes is similar to their *B*,*O*-congeners, except for the fact that the borylation step for configurationally stable 1,4-azaborinine helicenes is typically performed *via* lithium–halogen exchange (Cl or Br), while achiral DABNA derivatives could also be synthesized *via* directed *ortho*-lithiation.^67^ Accordingly, Yasuda prepared carbazole-based 1,4-azaborinine 40*via* lithium–halogen exchange between dibromo-substituted precursor 39 and *n*-Buli (Scheme 7). The target compound, which can be considered as a structural analogue of DABNA with two diphenylamine units replaced by 3,6-di-*tert*-butylcarbazole (BCz) moieties, was isolated in a yield of only 5%, along with the regioisomeric product in a 7% yield.^68^ No data regarding the configurational stability of this double [6]helicene are provided. Nonetheless, it may be inferred from the number and type of the constituent rings that its diastereomerization barrier Δ*G*^‡^_dias_ cannot be particularly high. In contrast, double [7]helicenes 41a–c (Scheme 7), consisting of a B-π-B and two N-π-N motifs in a *para* arrangement around the central benzene ring, exhibit an excellent configurational stability (see Section 3.1), which allowed their resolution into (*P*,*P*)- and (*M*,*M*) enantiomers by HPLC on a chiral stationary phase.^69^ These compounds, were synthesized independently in a similar manner by Zhang and Duan^70^ and the Wang group.^69^ Their procedures, differing in the solvent (*tert*-butylbenzene or *o*-DCB), the excess of reagents, reaction temperature and time provided the target compounds in moderate yields (23–53%).

Thus, from an overall point of view, the methods for the preparation of 1,4-oxaborinine and 1,4-azaborinine helicenes primarily depend on the one-pot borylation, which includes directed either *ortho*-lithiation or lithium–halogen exchange with *n*- or *t*-BuLi, a subsequent reaction with BBr_3_, and C–H borylation in the presence of a bulky amine. At least one of these steps is carried out at elevated temperature between 100 and 180 °C, which requires a certain stability of the intermediates and the target compounds under these rather harsh conditions.

Owing to promising optical properties for (CP)-OLEDs, this type of molecular design has become incredibly popular in recent years. Quite remarkably, from a relatively narrow set of building blocks and synthetic transformations, a large variety of chromophores have been synthesized with *B*,*B* or *N*,*N* in *para* arrangement, with several examples of helically chiral (and configurationally stable) dyes (see Section 3.1).

### 2.3. 1,2-Oxa-, 1,2-azaborinine helicenes and helicenes with O–B–O and N–B–N motifs

**Scheme 8:**
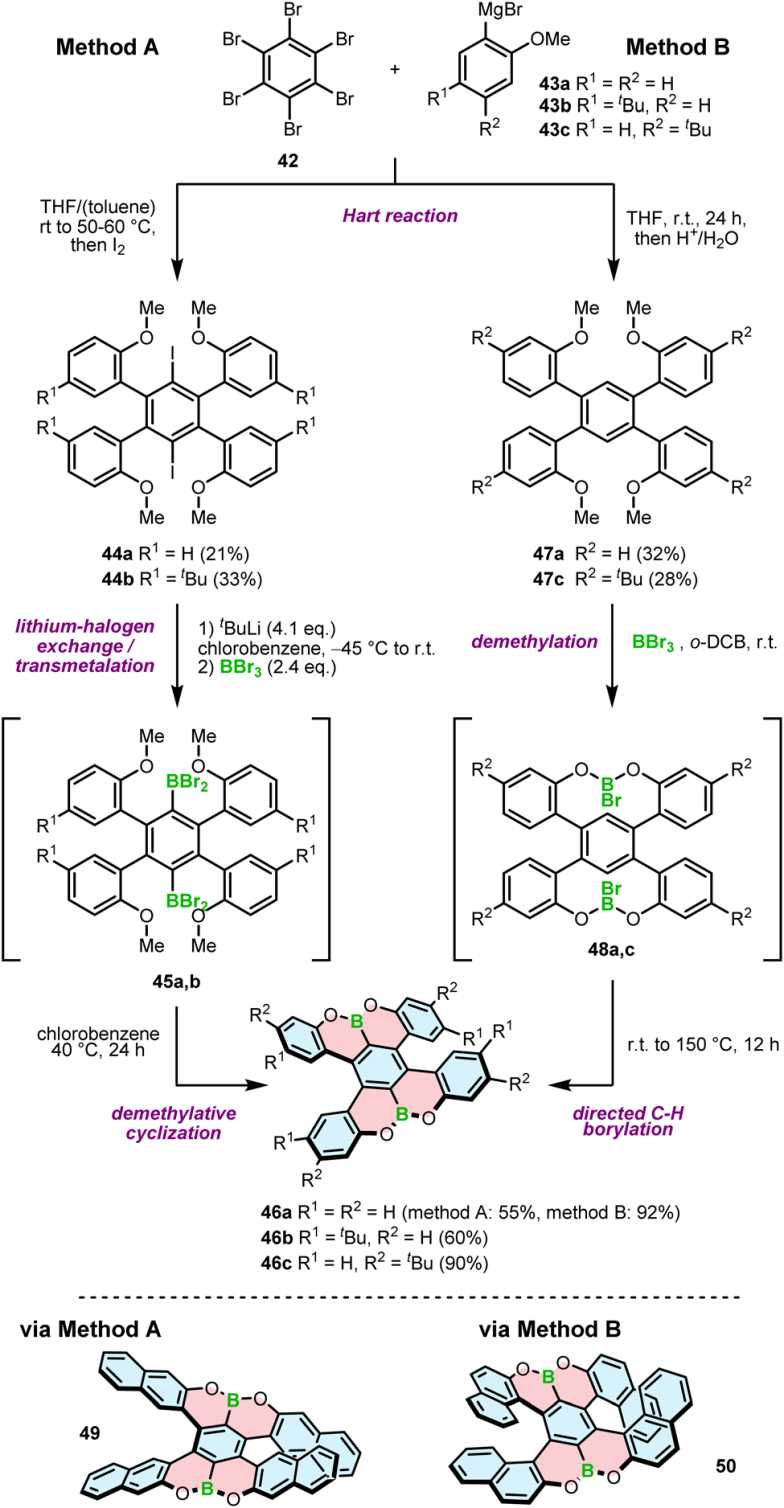
Synthetic strategy to O–B–O helicenes *via* demethylative cyclization. Method A: *via* lithium–halogen exchange; method B: *via* directed C–H borylation.

In contrast to the 1,4-oxaborinine derivatives, the syntheses of compounds containing O–B and O–B–O motifs usually involve demethylative C–H borylation. The role of BBr_3_ in these reactions is twofold. First, it cleaves the methyl ether, and second, it serves as a source of boron in the borylation reaction. This general approach was used independently by Feng and Müllen^73^ and the Hatakeyama group^71^ to develop double [5]helicenes 46a–c with a O–B–O motif. The synthesis reported by Hatakeyama started with the Hart reaction between hexabromobenzene (42) and arylmagnesium bromides 43a,b, followed by quenching with iodide. The presence of iodide in intermediates 44a,b invokes the following steps, that is, lithium–halogen exchange with *n*-BuLi, trapping of aryllithium with BBr_3_ and a consecutive demethylative ring closure at 40 °C (method A, Scheme 8). The reaction proceeds *via* intermediate 45a,b, the product of transmetalation with BBr_3_, and is accomplished by the subsequent formation of two B–O bonds to afford 46a and 46b in yields of 55% and 60%, respectively.^71^ A somewhat lower yield (43%) was obtained for double [5]helicene 49 with four linearly annelated benzene rings increasing the steric hindrance at the terminal positions. Here, the lithiation step was performed with *t*-BuLi.^72^ An alternative approach toward such double [5]helicenes, reported by Feng an Müllen, was based on halogen-free intermediates 47a,c and therefore, consisted of tandem demethylation with BBr_3_ to produce possible intermediate 48a,c and electrophilic C–H borylation at 150 °C in *o*-dichlorobenzene (*o*-DCB) (method B, Scheme 8). Target helicenes 46a and 46c were isolated in excellent yields of 90–92%.^73^ In general, the latter method appears to be higher-yielding and simpler, as there is no need for the installation of a halogen substituent on the precursor and hence, using a lithium–halogen exchange protocol to introduce a boron atom. On the other hand, the borylation step is performed under harsher conditions, which may possibly be problematic for more labile groups. The same approach was used to synthesize helically extended derivative 50 with the borylation carried out even at a higher temperature (180 °C). The yield of the target double [7]helicene was only 7%, considerably lower than that of its shorter congener, presumably due to steric strain that impeded the ring closure.^74^

**Scheme 9:**
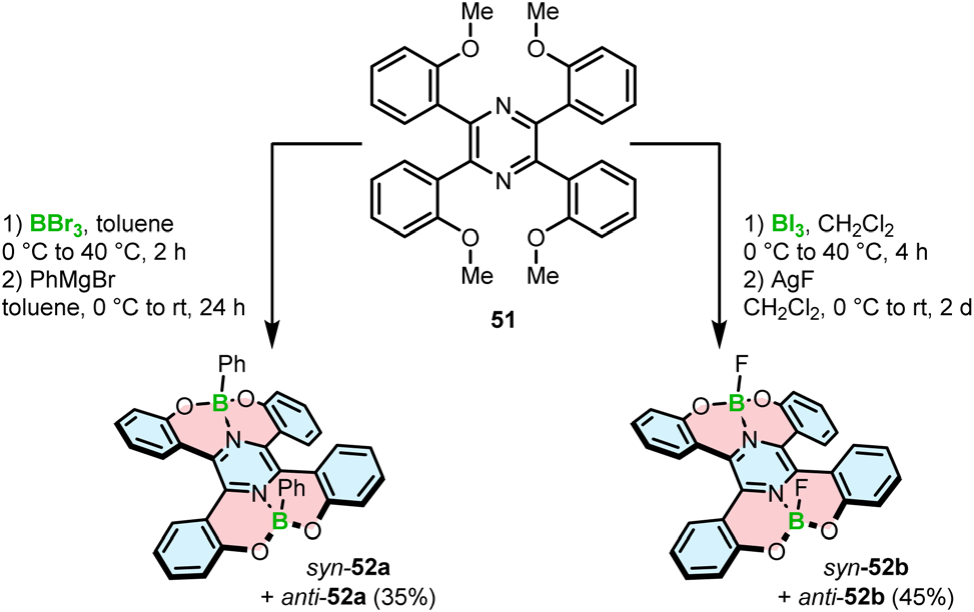
Synthesis of four-coordinate O–B–O helicenes.

Demethylative cyclization was also applied to prepare four-coordinate boron double [5]helicenes 52a,b containing two O–B–O motifs (Scheme 9). The compounds structurally resemble 46a with the difference that the central benzene ring is replaced with pyrazine.^75^ This seemingly little variation has tremendous implications for the construction of boracycles. Demethylation is accompanied by the smooth formation of two boron–nitrogen dative bonds in the last step, as opposed to the energetically more demanding C–B bonds. Therefore, the reactions either with BBr_3_ or more electrophilic BI_3_ could be carried out under mild conditions (at 40 °C *vs.* 150 °C for 46a). Similar to the synthesis of azaboroles, the synthesis was accomplished by the ligand exchange with Grignard reagent and AgF, respectively, to produce 52a and 52b in 35% and 45% yields. It is noteworthy that these compounds exhibit considerable chemical stability against acids and bases with no decomposition observed upon treatment with 0.5 N HCl or NaOH aqueous solutions.

**Scheme 10:**
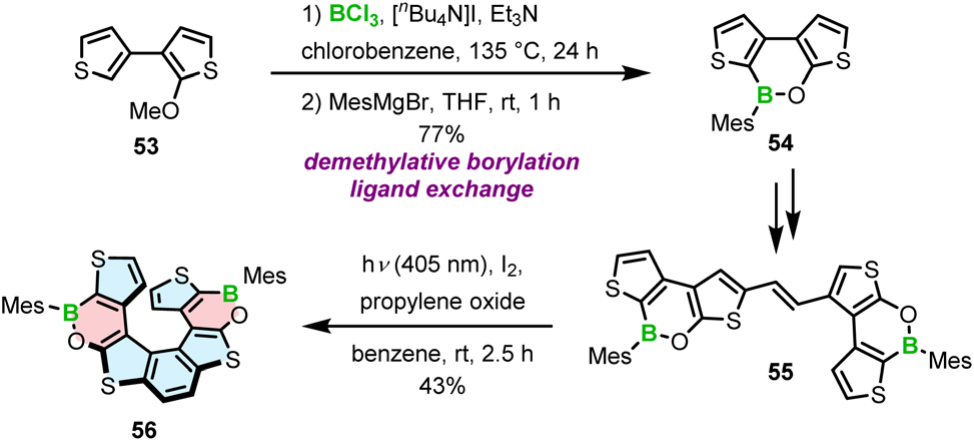
Synthesis of a B–O based helicene by postfunctionalization of a boracycle.

The strategy chosen by Licandro and Wagner to construct borathia[7]helicene 56 with two B–O bonds envisaged the formation of the boracycles prior to the formation of the helicene (Scheme 10).^76^ Thus, the three-ring system 54 was assembled *via* demethylative borylation of 53 with BCl_3_ in the presence of [*n*-Bu_4_N]I, and Et_3_N at an early stage of the synthesis. To sterically protect the tricoordinated boron atom, the chloride ligand was replaced with the bulky Mes in the following reaction, thus combining two approaches to stabilize boron, *i.e.* kinetic stabilization and electronic stabilization through donation of electron density from the neighboring oxygen atom. This compound was then converted in two steps, including bromination and the Stille coupling with (*E*)-1,2-bis(tributylstannyl)ethene into 55. The final Mallory photocyclization produced helicene 56 in a satisfactory yield. In contrast to the cyclization of benzene rings, where the other reactive site had to be protected (*cf.* the synthesis of **5–7**), the ring closure of thiophene-substituted ethenes leads to a single product. An important difference in the synthesis of 56 compared to the syntheses of other B–O or B–N helicenes is that the construction of boracycles was accomplished at an early stage of the synthesis, so that B–O bond formation was not hindered by steric strain.

The synthetic strategies toward helicenes with B–N or N–B–N motifs are more versatile. In 2012, Hatakeyama and Nakamura reported the synthesis of azaboradibenzo[6]helicene 59*via* a tandem bora-Friedel–Crafts-type reaction.^77^ Compared to the synthesis of B–O helicene 35, the double N-directed C–H borylation was performed with less reactive BCl_3_ at a higher temperature (150 °C *vs.* 100 °C for 35) and required utilization of AlCl_3_ in the presence of 2,2,6,6-tetramethylpiperidine (TMP).

**Scheme 11:**
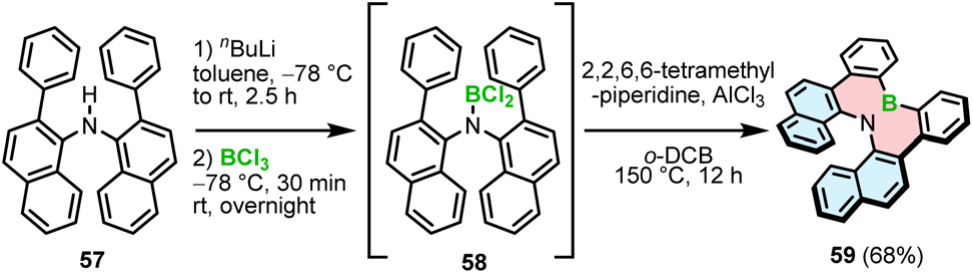
Synthesis of 1,2-azaborinine helicene 59.

This step was preceded by deprotonation of the NH functionality with *n*-BuLi. The target helicene with a boron–nitrogen covalent bond of 1.448(3) Å, indicating the strong π-interaction, was isolated in a 68% yield (Scheme 11).

**Scheme 12:**
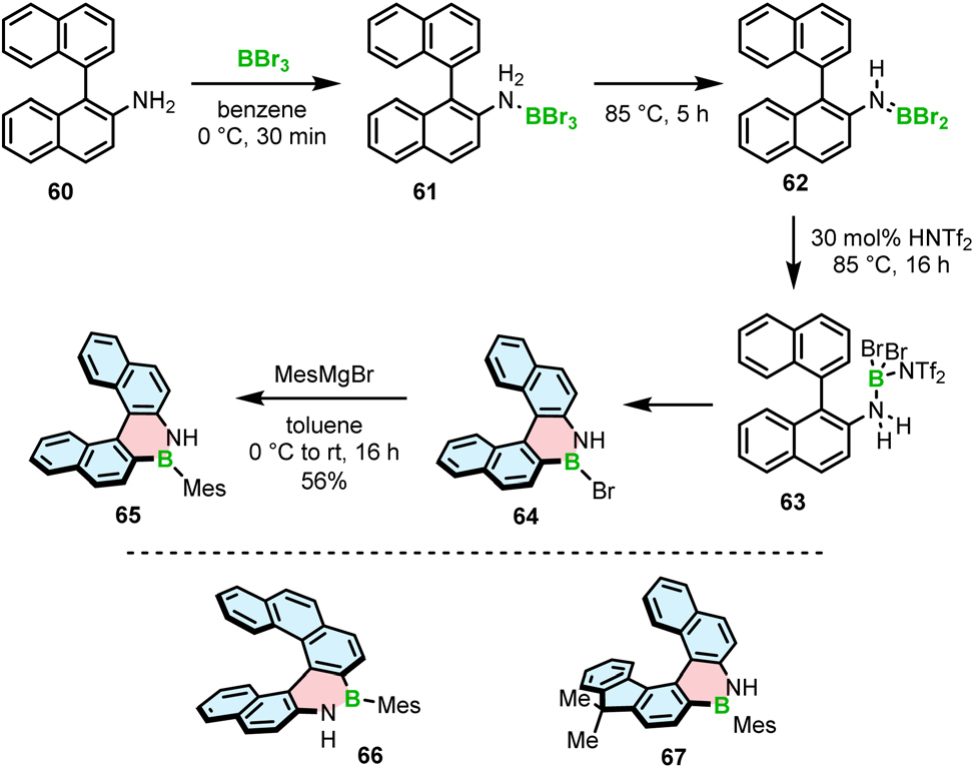
Synthesis of 1,2-azaborinine helicenes by electrophilic borylation.

The Ingleson group, with extensive expertise in the electrophilic borylation of various heterocycles,^78–81^ and our group reported the synthesis, resolution and characterization of the (chir)optical properties of the first configurationally stable core-non-extended [5]- and [6]helicenes 65–67 embedding an azaborine ring, where the B–N bond is located on the outer helicene rim.^82^ The attempts to prepare 65 using BCl_3_/AlCl_3_ under forcing conditions (up to 175 °C) or BBr_3_ at 75–150 °C either failed or the conversion was relatively low (<30%). It is worth noting that these conditions are suitable for the synthesis of simple planar compounds, again indicating the challenge of preparing the strained derivatives using existing synthetic protocols. The improved N-directed C–H borylation of 1,2-BN helicene 65 involves the generation of aminoborane 62 from the adduct with BBr_3_ at 85 °C, protonation of this species with bistriflimidic acid (HNTf_2_), leading to the formation of helicene 64 (*via* reactive species 63) that is directly reacted with 2-mesitylmagnesium bromide to afford 65 in a 56% yield (Scheme 12). The increased electrophilicity of borenium cations resulting from aminoborane protonation, compared to the activation of the latter species with BBr_3_, is responsible for the greater efficiency of this protocol. This HNTf_2_ mediated C–H borylation methodology allowed to access even more strained [6]helicenes 66 and 67 in 61% and 67% yields. Alternatively, the formation of aminoborane 62, and in turn the reactive borenium intermediate, can be executed by adding Me_3_SiNf_2_ that is generated *in situ* from HNTf_2_ and portionwise added PhSiMe_3_. The reaction in this case can be carried out at room temperature to provide the target bench stable compounds in 47–76% yields after substitution with MesMgBr. Phenyl derivative of 65 also was synthesized in a satisfactory yield (34%) using this protocol. However, the less bulky Ph did not provide sufficient kinetic protection and the compound was prone to decomposition. Mesityl-substituted helicene 65, on the other hand, could be readily converted into the *N*-methyl derivative using MeI and potassium bis(trimethylsilyl)amide (KHMDS).

**Scheme 13:**
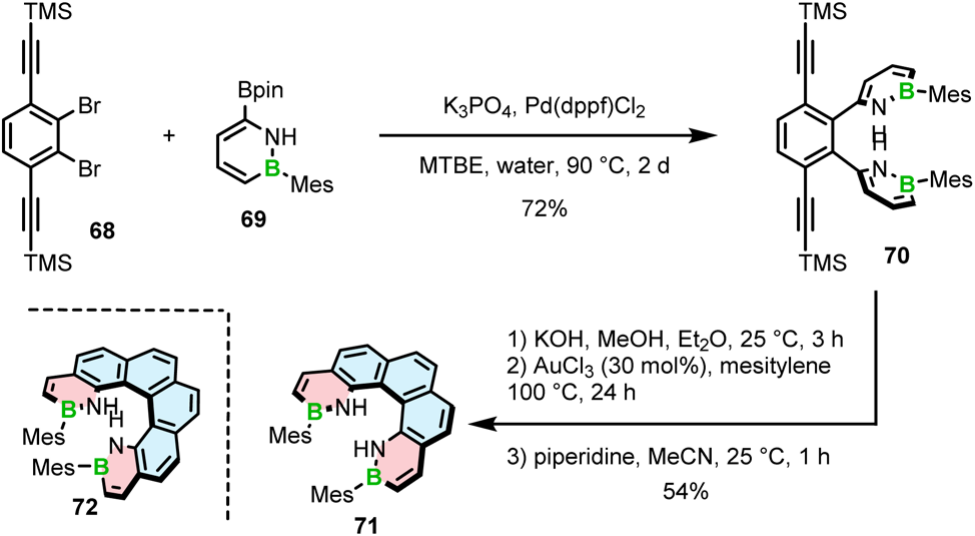
Synthesis of 1,2-azaborinine helicenes with two 1,2-azaborinine rings.

Only recently, Staubitz reported another approach toward core non-extended azaborine helicenes.^83^ As opposed to 65–67, the B–N bonds in compounds 71 and 72, consisting of two terminal azaborine rings, were placed on the inner helicene rim. The synthesis relied on the Suzuki coupling of Mes-substituted azaborine 69 with TMS-ethynyl-substituted dibromides (*e.g.*68), followed by double intramolecular cyclization (Scheme 13).

The last step proved challenging due to the competition between the 5-*exo-dig* and 6-*endo-dig* cyclization, which contrasts with the synthesis of other BN-doped PAHs where the latter pathway prevails.^84^ The identified conditions favoring the closure of the six-membered rings involved deprotonation with KOH and a subsequent cyclization with either AuCl_3_ at 100 °C or [(*p*-cymene)RuCl_2_]_2_ and AgSbF_6_ at 170 °C to produce [5]- and [6]helicenes 71 and 72 in 54% and 11% yields, respectively. Piperidine was added in the last step to facilitate the separation of the target helicenes from the resulting Michael adducts produced from the *exo*-cyclization products.

**Scheme 14:**
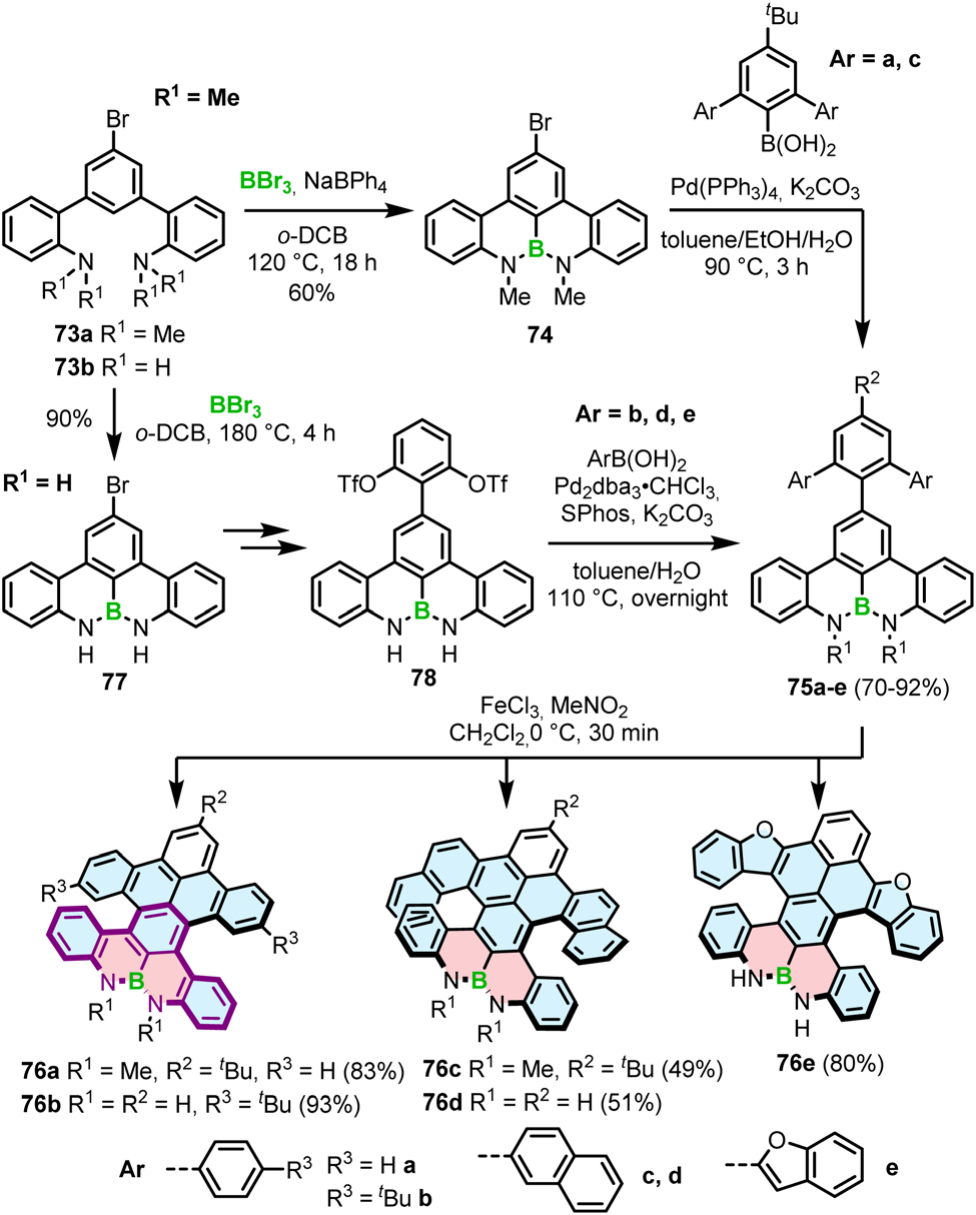
Synthesis of helicenes with a N–B–N motif at the zigzag edge.

Interestingly, a demethylative cyclization was applied not only to create B–O helicenes but also in the synthesis of helicenes with a N–B–N motif at the zigzag edge (Scheme 14). 73a, obtained by methylation of 73b, was converted into 74*via* borylation with BBr_3_ at 120 °C in the presence of NaBPh_4_, a non-coordinative Brønsted base.^85^ This key intermediate was cross-coupled with the aryl boronic acid building blocks to give 75a and 75c, which were submitted to the Scholl reaction with FeCl_3_ in the presence of MeNO_2_. Double [5]- and [6]helicenes 76a and 76c were obtained in yields of 83% and 49%, the latter being lower, though it is not clear if it is due to steric strain or regioselectivity issues.^86^ Alternatively, the synthesis of the key boron-containing building block can proceed in high yield directly from the corresponding NH derivative 73b*via* nitrogen-directed electrophilic borylation with BBr_3_ at 180 °C. The following Suzuki coupling of 77 with (2,6-dimethoxyphenyl)boronic acid and multiple functional group interconversion provided triflate derivative 78, which was cross-coupled with arylboronic esters to afford the helicenes precursors. The last step was carried out under the conditions reported earlier for 76a,c to produce 76b,d,e in 51–93% yields.^87^ Like in the case of B–O helicene 56, the formation of the boracycles was carried out early in the synthesis, thus not being affected by steric repulsion.

**Scheme 15:**
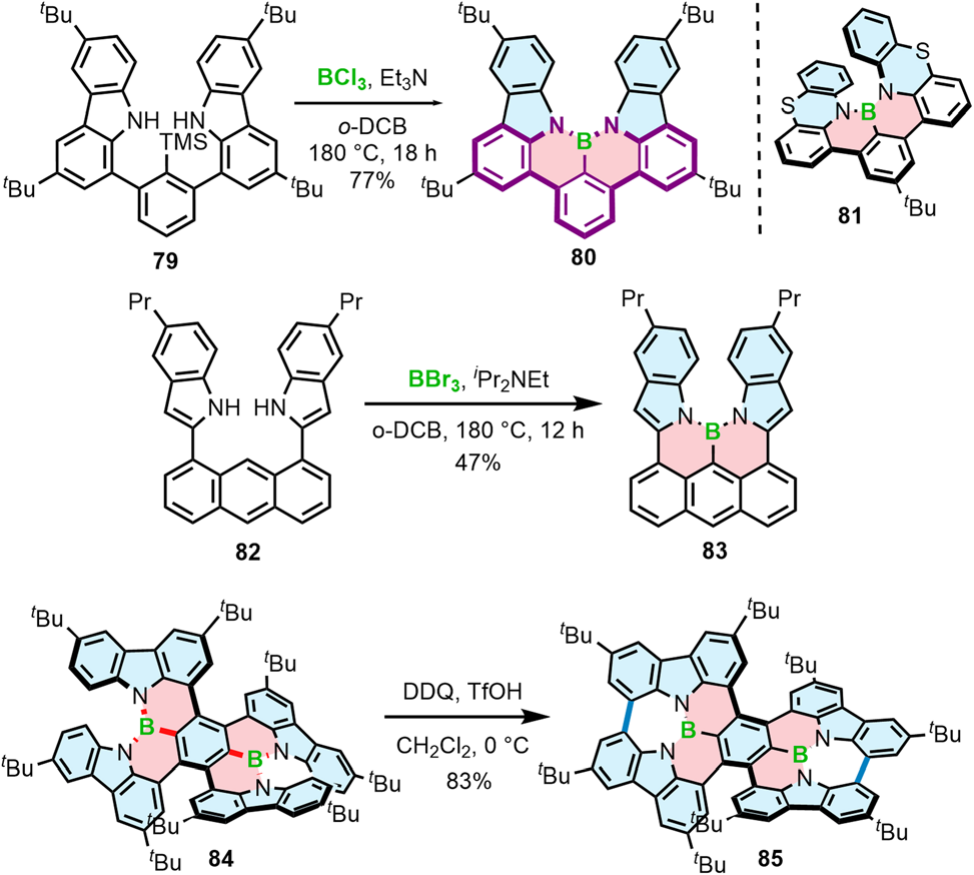
Synthesis of helicenes with a N–B–N motif on the inner helicene rim.

Different from the double helicenes reported by Zhang,^86,87^ the formation of the helicenes with an N–B–N motif on the inner helicene rim, whether configurationally stable of fluxional, takes place in the last step of the synthesis and therefore requires steric strain to be overcome. In principle, 76a and 80 consist of the same moiety (highlighted in purple) but the helical extension is realized through the C–N bonds of the boracycles in the latter case. The synthesis of 80 and 81 is performed *via* silicon–boron exchange between TMS-substituted precursors (*e.g.*79) and BCl_3_ accompanied by the formation of the B–N bonds in the presence of Et_3_N under forcing conditions (180 °C in *o*-DCB) (Scheme 15). The presence of the TMS group proved essential in reducing the reaction times and increasing their efficiency. These conditions failed however to prepare more rigid and strained 83, which was finally generated through the borylation of 82 with more reactive BBr_3_ in the presence of DIPEA in a 47% yield (Scheme 15).^88^

Recently, Yang capitalized on this method to prepare 84 with two N–B–N motifs in a 65% yield, where boron atoms were introduced using BBr_3_ as the boron source in the presence of DIPEA as the additive.^89^ In contrast, much lower yields were obtained when additives such as Et_3_N and TMP were used. The quadruple was then converted into double helicene 85*via* oxidative aromatic coupling using DDQ and TfOH in CH_2_Cl_2_ to form two NBN-doped heptagons (Scheme 15). Both compounds reveal excellent thermal stability in the solid state, however 84 tends to decompose in solution within a few hours. Higher stability of 85 can likely be attributed to its higher rigidity, which prevents it conformational distortion upon interaction with a nucleophile.

### 2.4. Helically chiral compounds doped with boron only

Introduction of boron into π-conjugated systems produces materials with high electron affinity. It has been postulated that boron-containing PAHs could be used as n-type semiconductors in transistors or as acceptors in solar cells. While this has been demonstrated for various boron compounds that also contain other heteroatoms, such as nitrogen, PAHs consisting of only C and B typically exhibit p-type mobility. This is due to the fact that such structures constitute a synthetic challenge. Only a small number of achiral PAHs containing exclusively carbon and boron atoms in π-conjugated scaffolds have been characterized to date. Even less common are their helical derivatives. The synthesis of these types of compounds thus represents uncharted scientific territory.

**Scheme 16:**
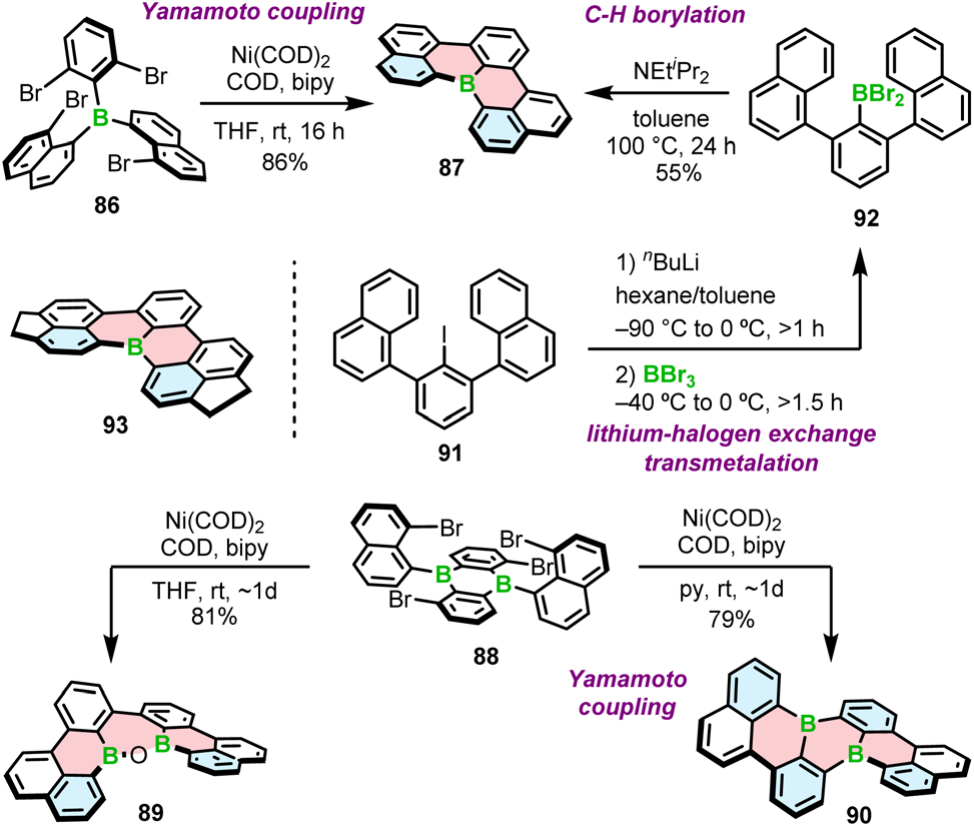
Synthesis of B-doped [4]helicenes and double [4]helicenes by Yamamoto coupling and lithium–halogen exchange/transmetalation/C–H borylation.

In 2015, Wagner and co-workers reported the highly fluorescent [4]helicenes through the intramolecular Yamamoto coupling of substituted triarylboranes.^90^ The reaction between tetrabrominated derivative 86 and four equivalents of bis(cycloocta-1,5-dien)nickel(0) (Ni(COD)_2_) in the presence of the COD ligand and 2,2′-bipyridyl in THF afforded target B-doped [4]helicene 87 in 86% yield (Scheme 16). In contrast, double iodide–lithium exchange and a subsequent intramolecular substitution of fluoride proved ineffective. Using nickel-mediated Yamamoto coupling of 88, Wagner and co-workers were also able to synthesize double [4]helicene 90. Surprisingly, the reaction outcome was strongly solvent-dependent. When the coupling was performed in THF and quenched with air, oxadiborepin 89, the rearrangement product was obtained as the main product in 81% yield (Scheme 16). On the other hand, Yamamoto coupling in pyridine as a solvent provided 90 in 79% yield. Mechanistic studies indicated that the rearrangement reaction is induced by the coordination of the hydroxide to the Lewis acidic boron. The presence of pyridine suppresses this process by blocking the coordination site.^91^

Nakayama and Hatakeyama proposed an alternative synthesis of 87 based on lithium–halogen exchange, subsequent transmetalation and C–H borylation with BBr_3_ in the presence of DIPEA (Scheme 16).^92^ In general, the synthesis of this compound is similar to that of [4]helicene 33 (Scheme 6), except that it proceeds *via* iodide 91. The latter compound is generated by selective lithiation of 1,3-dichlorobenzene (*m*-DCB) followed by reaction with 1-naphthylmagnesium bromide. The overall yield starting from *m*-DCB is comparable to that of the three-step method reported by Wagner (42% *vs.* 43%, respectively). As in the synthesis of 33, the presence and type of a base played a crucial role. In the absence of additives (DIPEA), the product was obtained in a yield of only 2%. According to the theoretical studies, the abstraction of a proton is exergonic and shifts the equilibrium to the product. This is also in agreement with high yield of 93 (82%) in the absence of amine (Scheme 16). Due to its poor solubility in toluene, 93 precipitates out of the reaction mixture, facilitating the formation of the product.

**Scheme 17:**
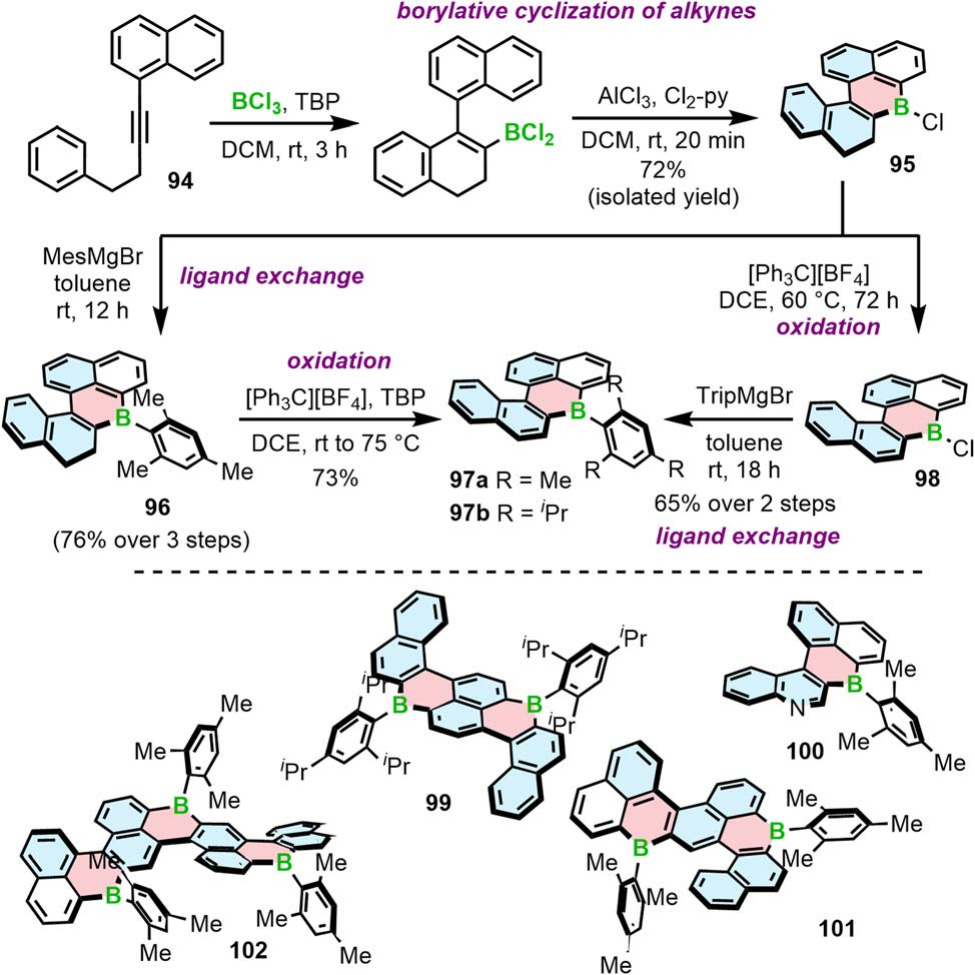
Synthesis of boron embedded [4]helicenes by borylative cyclization of alkynes.

Another approach to access B-doped systems consisting of angularly fused rings is based on borylative cyclization of alkynes with BCl_3_ in the presence of 2,4,6-tri-*tert*-butylpyridine (TBP) and subsequent intramolecular electrophilic C–H borylation induced by AlCl_3_/2,6-dichloropyridine (Cl_2_-py) to form a boracycle.^93^ This method, developed by Ingleson, was used to synthesize PAHs consisting of a single borahelicene as well as multiples assembled from two or three bora[4]helicene subunits (Scheme 17).^93–95^ The first step of this reaction sequence involves the activation of alkyne 94 with BCl_3_ and intramolecular S_E_Ar. The reaction proceeded cleaner in the presence of the hindered base.^93^ The following formation of the boracycle was performed using stoichiometric amount of AlCl_3_ and 2,6-dichloropyridine to give chloride 95 in 72%, sensitive to moisture. This compound was then converted into 96 with the mesityl group providing sufficient kinetic stabilization. 96 can be readily oxidized to 97a using [Ph_3_C][BF_4_]/TBP. Compound 97b could not be accessed in this way because the isopropyl groups are not inert under these conditions. Thus, 97b was synthesized *via* oxidation of 95 to 98 and *in situ* addition of the corresponding Grignard reagent. Analogous approach was used to prepare double [4]helicene 99 starting from the corresponding dialkyne. The synthesis of unique B,N-doped 100 (Scheme 17) required certain modification, such as a greater excess of AlCl_3_ and the use a different organometallic reagent (ZnMes_2_) due to the presence of *N*-tosyl group in its precursor, which can be cleaved with MesMgBr.^94^

97a can also serve as a starting material for the iterative synthesis of PAHs 101 and 102 containing two or even three boron atoms. Accordingly, it can be regioselectively brominated with NBS in the presence of a catalytic amount of HCl, enabling Sonogashira coupling/cyclization/aromatization to afford 101. This reaction sequence can be repeated using 101 as the starting material to provide ribbon 102 (Scheme 17).^95^

**Fig. 2:**
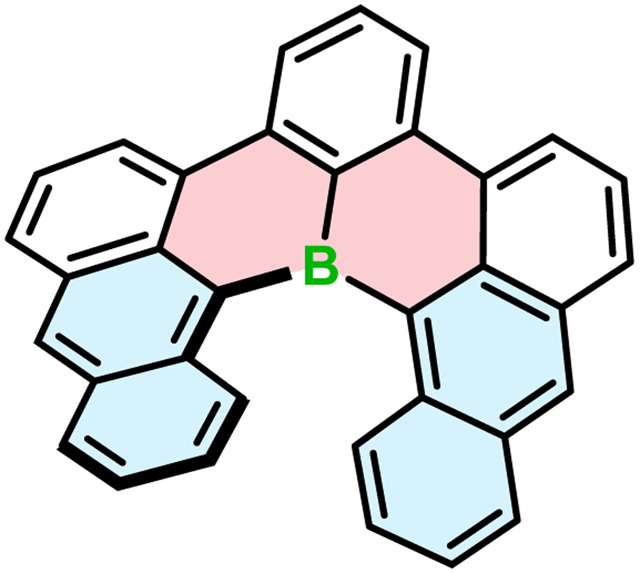
The structure of configurationally stable helicene 103 consisting of only carbon and boron atoms.

All the examples presented above contain [4]helicene motifs. Importantly, the presence of only four angularly fused six-membered rings does not provide sufficient configurational stability. Even though these compounds are helically chiral, their (*P*)*-* and (*M*)-enantiomers rapidly interconvert in each other. Therefore, they cannot be used as chiral materials. Only recently, Würthner and co-workers presented the first example of boron-doped configurationally stable helicene. This π-extended [6]helicene 103 (Fig. 2) was prepared from the iodo-teraryl with the anthryl moieties (in place of two naphthyl in 91) following the strategy applied by Hatakeyama for the preparation of 87. The compound was obtained in lower yield (17%), most probably due to the increased steric congestion. It showed excellent stability in the solid state under ambient conditions, when exposed to light and at high temperatures. However, it tends to decompose slowly when dissolved in CHCl_3_.^44^

### 2.5. BODIPY-based helically chiral dyes and related systems

Due to their unique optical properties, chemical versatility and stability, BODIPY dyes are utilized in various technological and bioapplications such as sensors, solar cells or OLEDs.^96,97^ Their high luminescence quantum yields, excellent photostability and the ability to readily tune their absorption and emission made them particularly attractive for use as fluorescent dyes in bioimaging.^98,99^ The synthesis of chiral derivatives opened new perspectives for this well-established class of compounds, such as applications in chiral sensing, asymmetric catalysis or as CPL-materials for advanced technologies in photonics and optoelectronics. To introduce chirality into BODIPYs, different strategies haven been employed, mainly exploiting the concept of axial chirality. For instance, axially chiral molecules with twisted and sometimes helical conformations have been achieved through the introduction of aryl moieties with hindered rotation into unsymmetrical BODIPYs or the attachment of groups such as chiral binaphthyl or 1,2-diphenyl-1,2-ethanolamine.^100,101^ Additionally, the BODIPY can be either attached to the periphery of a helicene, as shown by recent examples,^102–104^ or directly incorporated into the helical backbone, which is addressed in this section.

**Scheme 18:**
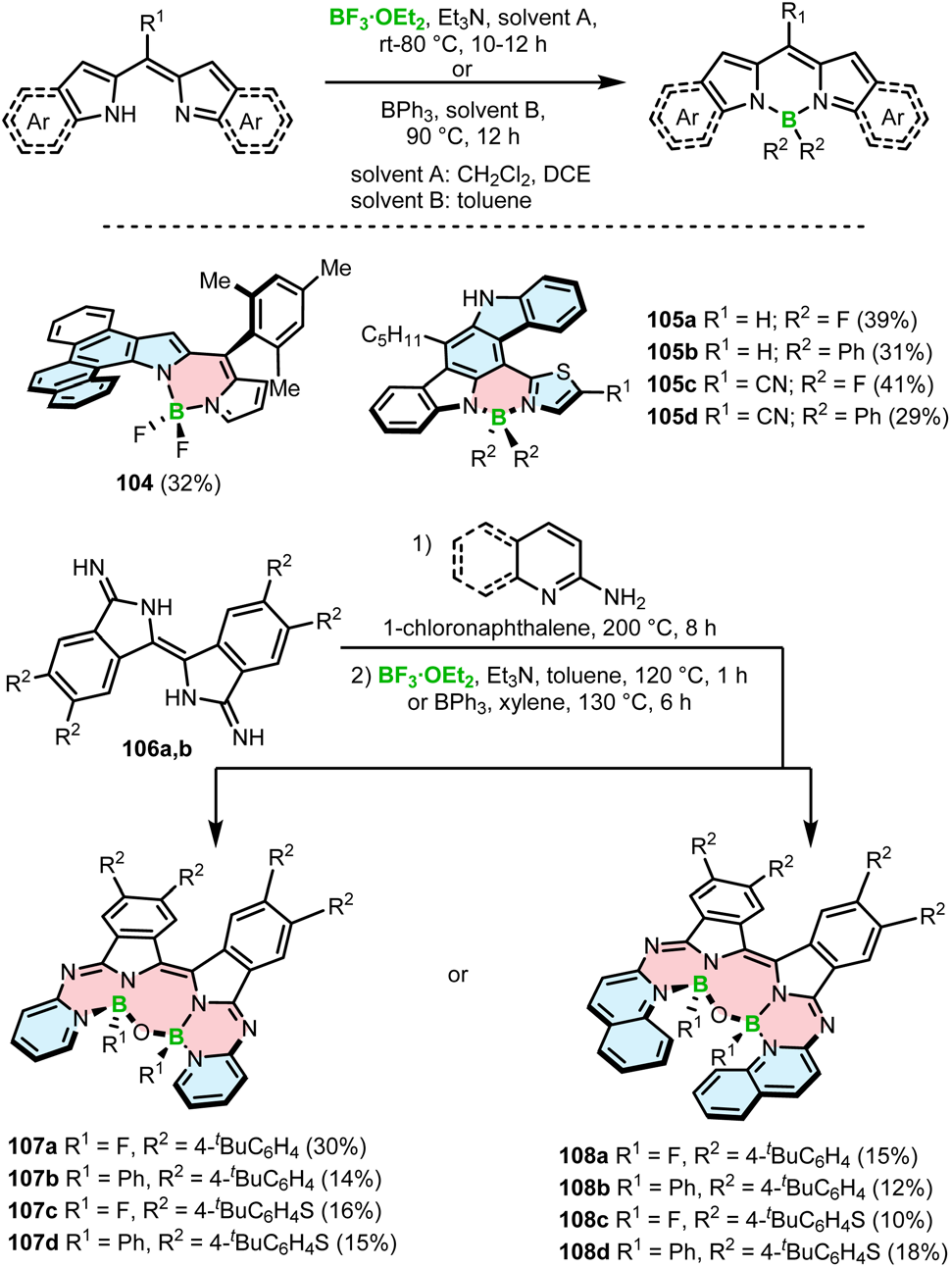
Synthesis of BODIPY–helicenes *via* borylation with BF_3_·OEt_2_ or BPh_3_ in the presence of an amine in the final step.

The synthesis of BODIPY-based helicenes is commonly achieved using the method developed for non-helical congeners, which involves borylation of dipyrromethane derivatives with BF_3_·OEt_2_ or BPh_3_ in the presence of Et_3_N in the final step. Using this general approach, Kawamata and Hasobe prepared helicene-like BODIPY 104 with the BF_2_ unit on the inner helicene rim in a yield of 32% (Scheme 18). Single crystal X-ray analysis confirmed a repulsion between the fluorine atom and the inner hydrogen atom of the terminal benzoid ring, resulting in a torsion angle of 35° between the BODIPY and chrysene moieties.^105^

In a similar way, Mula and co-workers synthesized the series of hetero[5]helicenes 105a–d bearing fluoride or phenyl substituents on boron on the outer helicene rim (Scheme 18).^106^ These BODIPY analogues, where one pyrrole ring was replaced by the thiazole ring, were isolated in yields ranging from 29 to 41%. The reactions with BPh_3_ led to somewhat lower yields compared to BF_3_·OEt_2_. Although both types of BODIPY–helicenes adopt a helical geometry, no information is provided regarding their configurational stability. In contrast, β-isoindigo-based helicenes 107 and 108 with two aza-BODIPY subunits exhibit sufficiently high enantiomerization barriers, which permitted their resolution into *P*- and *M*-enantiomers by HPLC. These compounds with two boron centers were prepared by condensation of diimino-β-isoindigo derivatives 106a,b with 2-aminopyridine or 2-aminoquinoline followed by borylation with BF_3_·OEt_2_ or BPh_3_ in the presence of Et_3_N to provide 107a–d and 108a–d in yields of 10–30% (Scheme 18). The O–B–O bridge connecting both arms of the molecule and giving rise to the seven-membered central ring results from hydrolysis of the C–B or C–F bonds by residual water in the solvent. The compounds exhibit excellent air-stability in the solid state, displaying no signs of decomposition even after being stored for six months.^107^

**Scheme 19:**
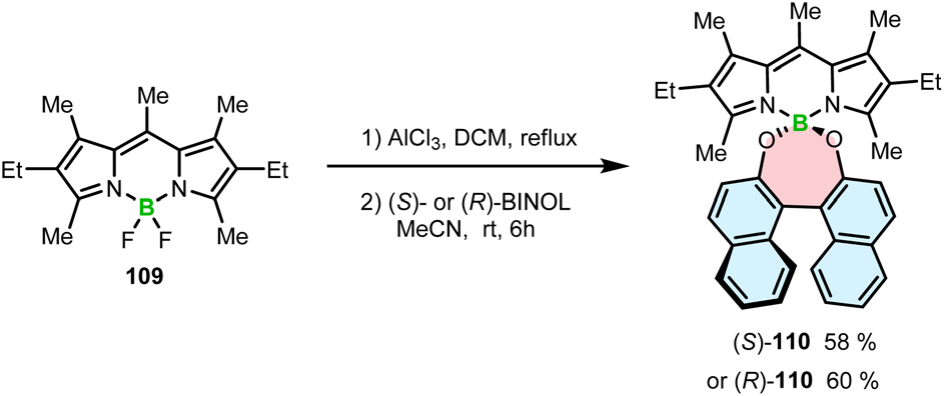
Synthesis of BINOL-BODIPYs.

High chemical stability of BODIPYs allows their post-modification, as demonstrated by nucleophilic substitution of fluoride by hydroxyl groups.^108^ This strategy toward axially chiral BODIPYs involves the assembling of an achiral BODIPY chromophore with a chiral BINOL moiety in a spiro arrangement. Thus, BODIPY 109 was reacted with (*S*)- or (*R*)-BINOL in the presence of Lewis acidic AlCl_3_. The role of AlCl_3_ was to activate the B–F bonds prior to the substitution with the OH groups to provide (*S*)- and (*R*)-110 in 58 and 60% yields, respectively (Scheme 19). This work demonstrates a simple way to produce helical boron compounds from commercially available materials in a single reaction step.^109^ However, the helical conformation is induced by the BINOL moieties, whereas a BODIPY chromophore is not part of the helical framework.

**Scheme 20:**
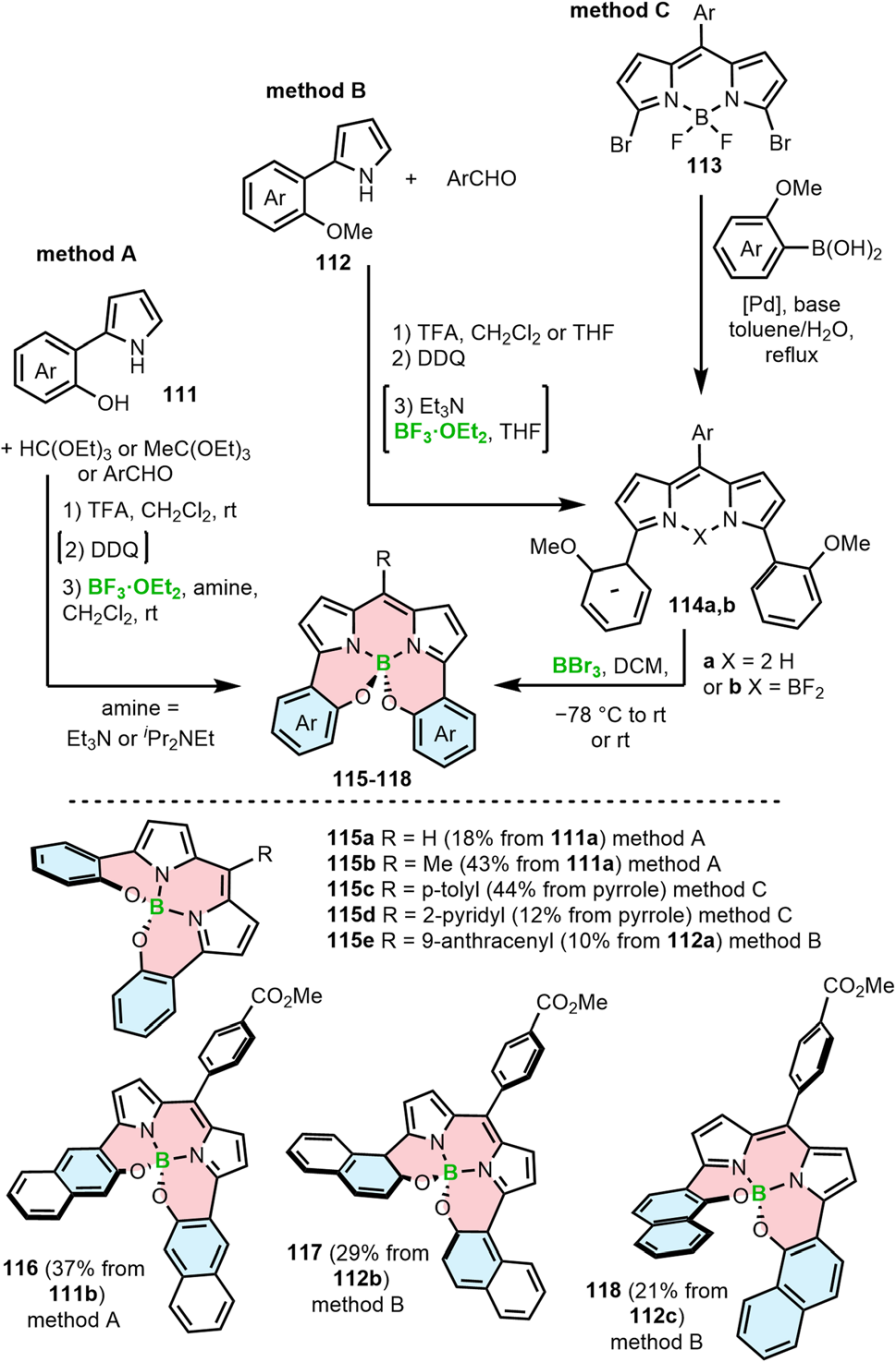
Synthesis of BODIPY–helicenes by nucleophilic substitution with hydroxy-substituted aryls or demethylative borylation in the key step.

A related strategy leading to the incorporation of a BODIPY moiety into the helical framework involves the reaction of hydroxyl groups attached to the BODIPY α-aryl substituents. This can be realized *via* three general approaches (Scheme 20). In method A, aryl-substituted pyrrole 111 with a free hydroxyl group is reacted with triethyl orthoformate, orthoacetate^110^ or an aryl aldehyde.^111^ In the latter case, the acid-catalyzed condensation is followed by oxidation with DDQ. The intermediate alkyl or aryl-substituted BODIPYs are formed *via* borylation with boron trifluoride diethyl etherate. The synthesis is accomplished by the substitution of fluoride with the hydroxyl groups to provide *O*-BODIPYs (*e.g.*115a,b and 116). In Method B, methoxy-substituted aryl 112 is used as a starting material. A similar reaction sequence leads to BODIPY 114b (synthesis of 115e)^112^ or to dipyrromethene 114a when the borylation with BF_3_·Et_2_O is skipped (synthesis of 117 and 118).^111^ In the final step the free OH groups are liberated upon treatment with BBr_3_ which then form the target *O*-BODIPYs. Alternatively, the compounds (*e.g.*115c,d) can be prepared by cross-coupling of dibrominated BODIPY 113 with methoxy-aryl boronic acids, followed by the cleavage of the methyl ether and the intramolecular substitution of fluorides with the liberated OH groups (method C).^110^ The use of MeO-aryls proved to facilitate the overall synthesis by preventing the unwanted side-reactions of the hydroxyl groups, hindering the preparation of the required intermediates or the target *O*-BODIPYs (*vide infra*). Similar approaches have been used to synthesize so-called aza-BODIPYs, *i.e.* the BODIPYs analogs where the carbon atom in the methene bridge is replaced by nitrogen.^113,114^

An unexpected result was obtained when dibromide-BODIPY 119 was cross-coupled with 2-hydroxyphenylboronic acid (120).

**Scheme 21:**
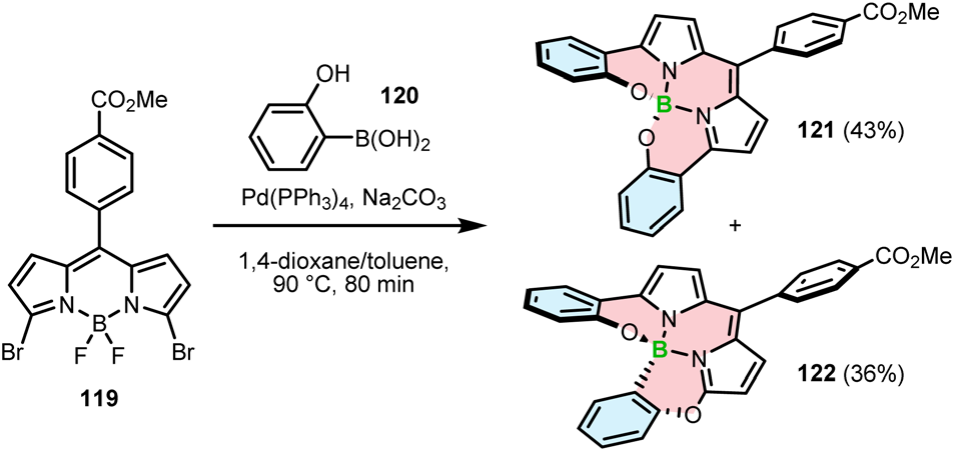
Synthesis of regioisomeric BODIPY–helicenes by Suzuki–Miyaura cross-coupling of a dibromo-BODIPY and 2-hydroxyphenylboronic acid.

In addition to the desired *N*,*N*,*O*,*O*-chelate 121 (43%), Hall and co-workers isolated *N*,*N*,*O*,*C*-chelate 122 in 36% yield (Scheme 21). The formation of regioisomeric 122 was rationalized by a reaction cascade involving the metathesis of BF_2_ with the boron atom of 120, the formation of the C–B bond by S_N_Ar, Suzuki coupling with a second equivalent of 120 and the final formation of the B–O bond. However, the detailed reaction mechanism has not been elucidated.^115^

## 3. Properties of chiral B-doped PAHs

### 3.1. Configurational stability as an important stereodynamic factor

Configurational stability of helicenes is an important stereodynamic feature that determines whether a given compound is suitable for use as a chiral material. It is believed that the minimal barrier required to resolve the enantiomers is *ca.* 22.7 kcal mol^−1^ (95 kJ mol^−1^) at 300 K.^116^ This value is however not sufficient from a practical point of view, as the compounds with such low barriers would racemize relatively fast at room temperature or at only slightly elevated temperatures. A high Δ*G*^‡^ is therefore a prerequisite for the use of helicenes in devices in order to take advantage of their chirality. More specifically, it must ensure that the compound does not racemize during device fabrication. For instance, it is required to enable thermal treatment of a compound or a stack, such as thermal evaporation and deposition under high vacuum or thermal annealing. The threshold Δ*G*^‡^ value for optoelectronic applications is *ca.* 35 kcal mol^−1^, but the exact value depends on the processing conditions and can be higher. The method of choice to study the *P*–*M* interconversion processes of helicenes depends on their Δ*G*^‡^ value. For a comprehensive overview of various methods, readers are directed to the tutorial article by Mayor.^117^

**Scheme 22:**
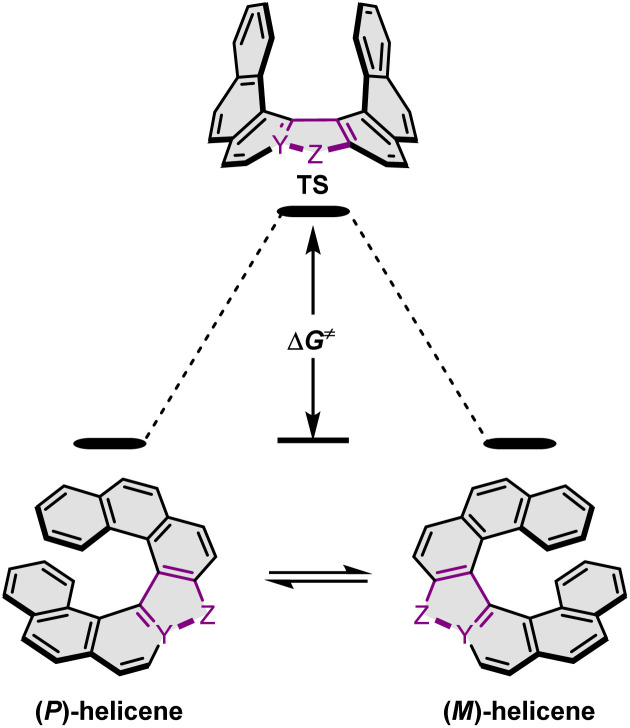
Enantiomerization of a helicene with a five-membered ring. Y and Z denote carbon or a heteroatom.

**Fig. 3:**
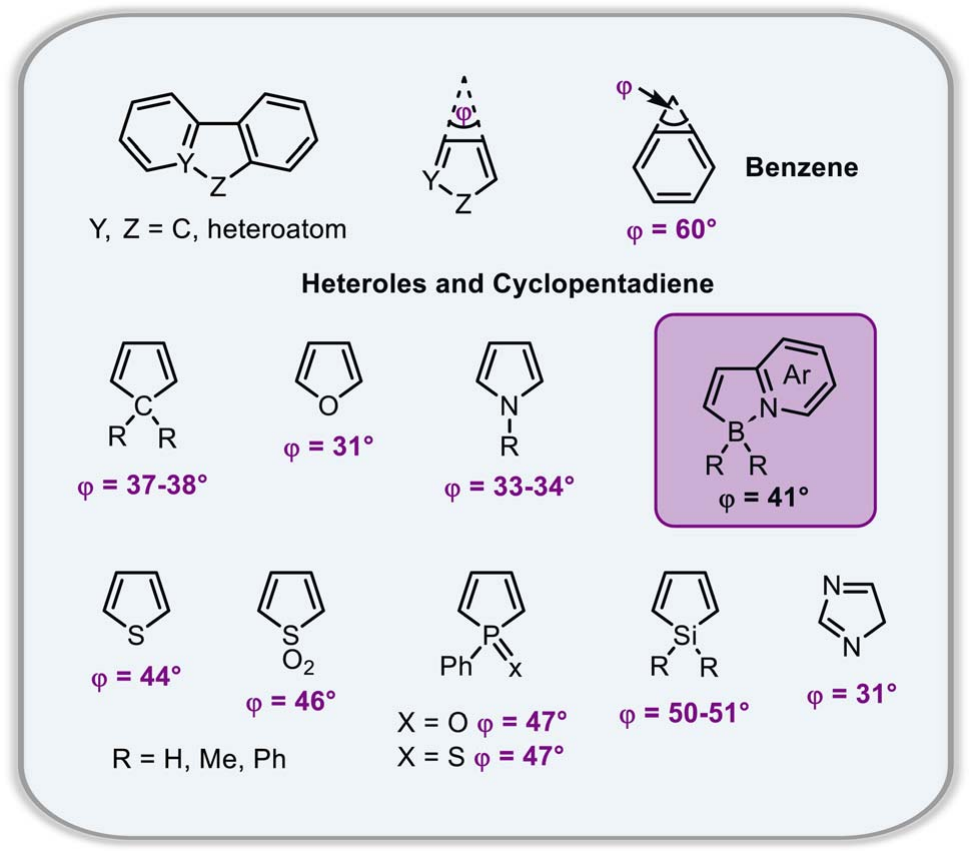
Wedge angles *φ* of cyclopentadiene and heteroles calculated at the B3LYP/def2-TZVP level of theory.

**Fig. 4:**
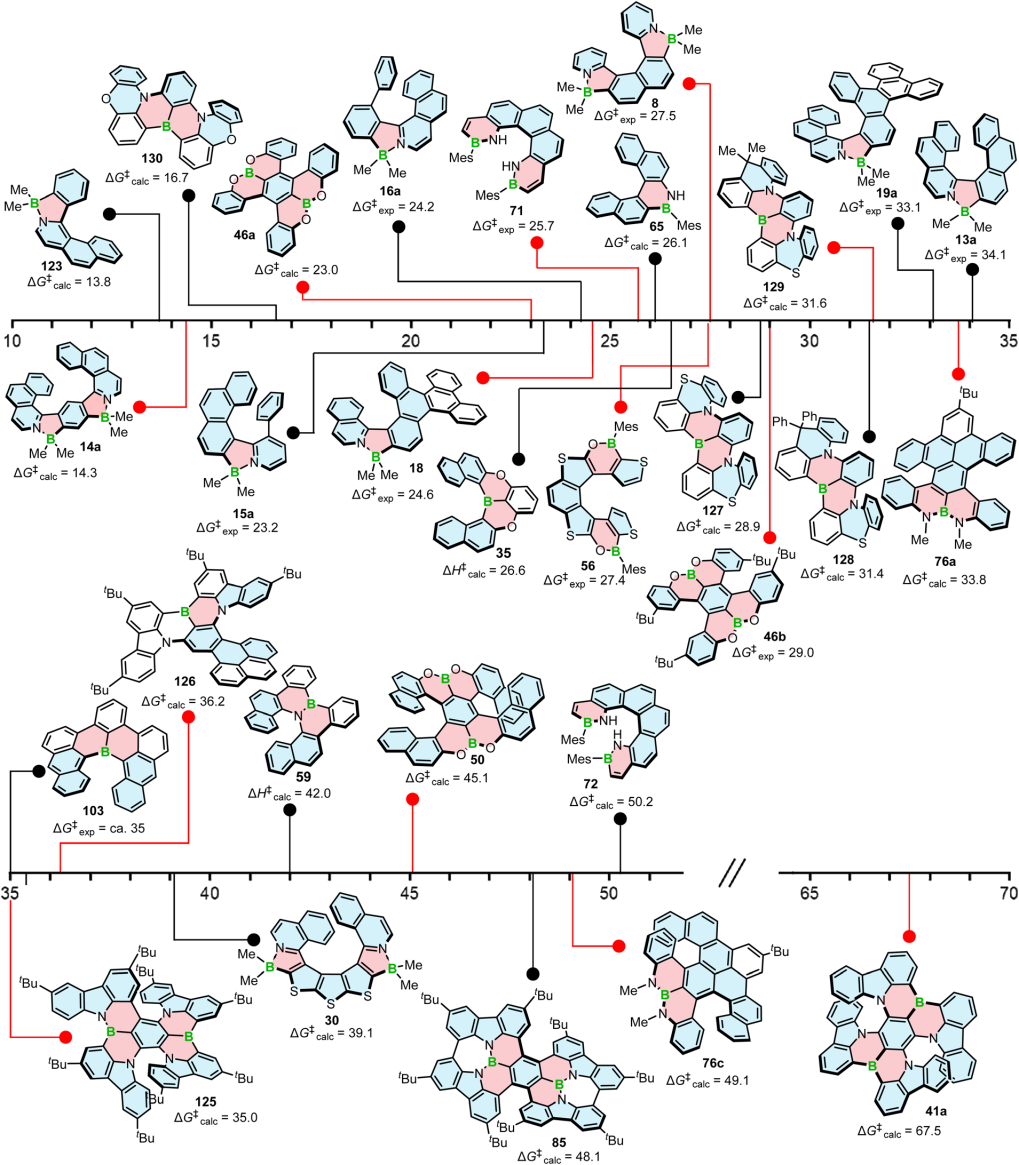
Configurational stability in kcal mol^−1^ of various boron helicenes. The experimental Δ*G*^‡^ value is given where available.

The *P*–*M* interconversion of carbo- and heterohelicenes with a single helical twist proceeds *via* one TS in which the outer rings at both ends of the molecule face each other (*e.g.* enantiomerization of a helicene with a five-membered ring, Scheme 22). The distortion of the molecule in TS depends on the steric congestion imparted by the terminal rings. For a smaller number of rings, it can be nearly planar. It holds true for carbo[4]helicene with six-membered benzoid rings. However, the inversion barrier in this case is extremely low and the racemate cannot be resolved into its enantiomers. Helical extension by only one benzene ring dramatically increased Δ*G*^‡^ to 23.9 kcal mol^−1^.^118^ It confers carbo[5]helicene with sufficient configurational stability to separate enantiomers, although the compound racemizes slowly at room temperature. A satisfactory barrier of 36.2 kcal mol^−1^ is reached for carbo[6]helicene.^119^ Thus, Δ*G*^‡^ of carbo[*n*]helicenes consisting of *ortho*-fused benzene rings steadily increases with the elongation of the helix, reaching a plateau for *n* = 7, 8, and 9. Theoretical studies of this class of compounds (where *n* ≤ 24) revealed that the *P*–*M* interconversion for *n* = 4–7 is a concerted process, while it follows a multi-step mechanism for *n* ≥ 8 *via* 2*n*-14 intermediates.^120^ Similarly, the increase in the number of angularly fused rings in heterohelicenes correlates with the increase in their configurational stability. It is important to note, however, that Δ*G*^‡^ is closely related not only to the number of constituent rings, but also to their type and hence, the change in the mechanism may occur for a different *n* number. The incorporation of five-membered rings into helicenes tends to lower their enantiomerization barriers due to a less favorable geometry of heteroles or cyclopentadiene. In addition, the loss in configurational stability depends on the type of atoms that form a given ring. Their radii determine the carbon–carbon, carbon–heteroatom or heteroatom–heteroatom bond lengths and thus the ring geometry, which in turn, determines the overlap of the two terminal rings. It can be quantitatively expressed by wedge angle *φ* between the two formal carbon–carbon or carbon–heteroatom double bonds (see Fig. 3). In principle, a larger *φ* value results in a stronger overlap of terminal rings and in turn, a stronger steric repulsion. For example, *φ* of 31° for a furan ring represents the lowest value among heteroles and carbocyclic rings listed in Fig. 3, indicating the strongest tendency of furan-based helicenes toward racemization. This was experimentally verified for helicene-like molecules bearing either furan, pyrrole or thiophene rings. Accordingly, a thermal treatment of a furan derivative in DMF led to the fastest decay of the CD effect.^121^ Furthermore, a [7]helicene consisting of one furan ring showed deterioration of the enantiomeric excess from initial 92% to 42% after 88 h of heating at 100 °C in toluene.^122^ Conversely, sila[7]helicenes with the highest *φ* value are expected to have the highest barriers to racemization among the five-membered derivatives. Indeed, no racemization occurred for a structurally similar dimethylsila[7]helicene that was kept at a temperature as high as 220 °C.^123^ Its Δ*G*^‡^_calc_ was calculated to be 37.4 kcal mol^−1^.^124^ Thus, *φ* serves as a good measure to evaluate the configurational stability of the corresponding helicenes, and hence these angles have often been reported. In addition, they were used to predict the geometry of circulenes.^125^ However, the published values have been measured in different ways, and in some cases derived for non-planar or helical structures. Therefore, for the purpose of this article, the wedge angles have been determined for the optimized geometries (B3LYP^126–128^/def2-TZVP^129–131^) of various planar three-ring systems, *i.e.* dibenzoheteroles and fluorene derivatives bearing various substituents on the central fusion atoms. The latter have only a minor effect on the geometry of the rings. Fig. 3 presents angles *φ* for various heteroles. The value for an azaborole is located somewhere between those for furan and silole and corresponds to the intermediate Δ*G*^‡^_exp_ value for azaborole [7]helicene 13a of 34.1 kcal mol^−1^ (at 180 °C),^55^ similar to the barrier of carbo[6]helicene. Thus, *φ* value of 41° translates to the excellent configurational stability of this compound. In contrast, 14a is conformationally labile. This helicene consisting of both angularly and linearly fused rings can be considered as a superposition of two azabora[5]helicenes with a joint benzene ring. DFT calculations (B3LYP-D3(BJ)/def2-SVP,^129–131^ CH_2_Cl_2_, PCM model) revealed that a combination of four benzene and one azaborole angularly fused rings is clearly insufficient to inhibit the *P*–*M* interconversion. The computed Δ*G*^‡^_calc_ (298 K) obtained for 123 (Fig. 4) was only 13.8 kcal mol^−1^. Notably, the barrier calculated for the rate-determining step for 14a was comparable (14.3 kcal mol^−1^).^55^ This indicates that the presence of the second benzoisoquinoline moiety, linearly fused to the [5]helicene core, results in a more complex *P*–*M* interconversion process (overall three TS), but exerts a negligible effect on the inversion barrier of the compound. Indeed, to ensure sufficient configurational stability, azabora[5]helicene has to be equipped with a bulky substituent at a sterically hindered position. This structural modification led to derivatives 15a,b and 16a,b that could be resolved into their enantiomers, although their Δ*G*^‡^_calc_ (B3LYP-D3(BJ)/def2-TZVP, CH_2_Cl_2_, PCM model) are relatively low (26.3–26.8 kcal mol^−1^). The barriers for 15a and 16a were also determined experimentally either by dynamic HPLC or thermal racemization experiment in *o*-DCB to give Δ*G*^‡^_exp_ of 23.2 kcal mol^−1^ (298 K), and 24.2 kcal mol^−1^ (323 K), respectively, at the lower limit of the feasible resolution.^57^ The lateral extension leads to a slight decrease in configurational stability compared to the non-extended derivatives. Accordingly, Δ*G*^‡^_exp_ of 19a was as high as 33.1 kcal mol^−1^ (443 K), while Δ*G*^‡^_exp_ of 18 was determined as 24.6 kcal mol^−1^ (338 K), and is therefore comparable with those of the phenyl-appended azaborole [5]helicenes.^56^ Interestingly, Δ*G*^‡^_exp_ of [6]helicene 8 with two azaborole rings falls in the same range (27.5 kcal mol^−1^ (351 K)).^54^

Δ*G*^‡^ calculated for azabora[9]helicene 30 of 39.1 kcal mol^−1^ (B3LYP-D3(BJ)/def2-TZVP, CH_2_Cl_2_, PCM model) is higher than that of 13a. However, the difference is not significant. This is due to the fact that five out of the nine rings are five-membered with less favorable geometry. Consequently, the enantiomerization of this compound proceeds *via* a single TS, whereas a multi-step process is already expected for carbo[8]helicene (see above).^63^

**Fig. 5:**
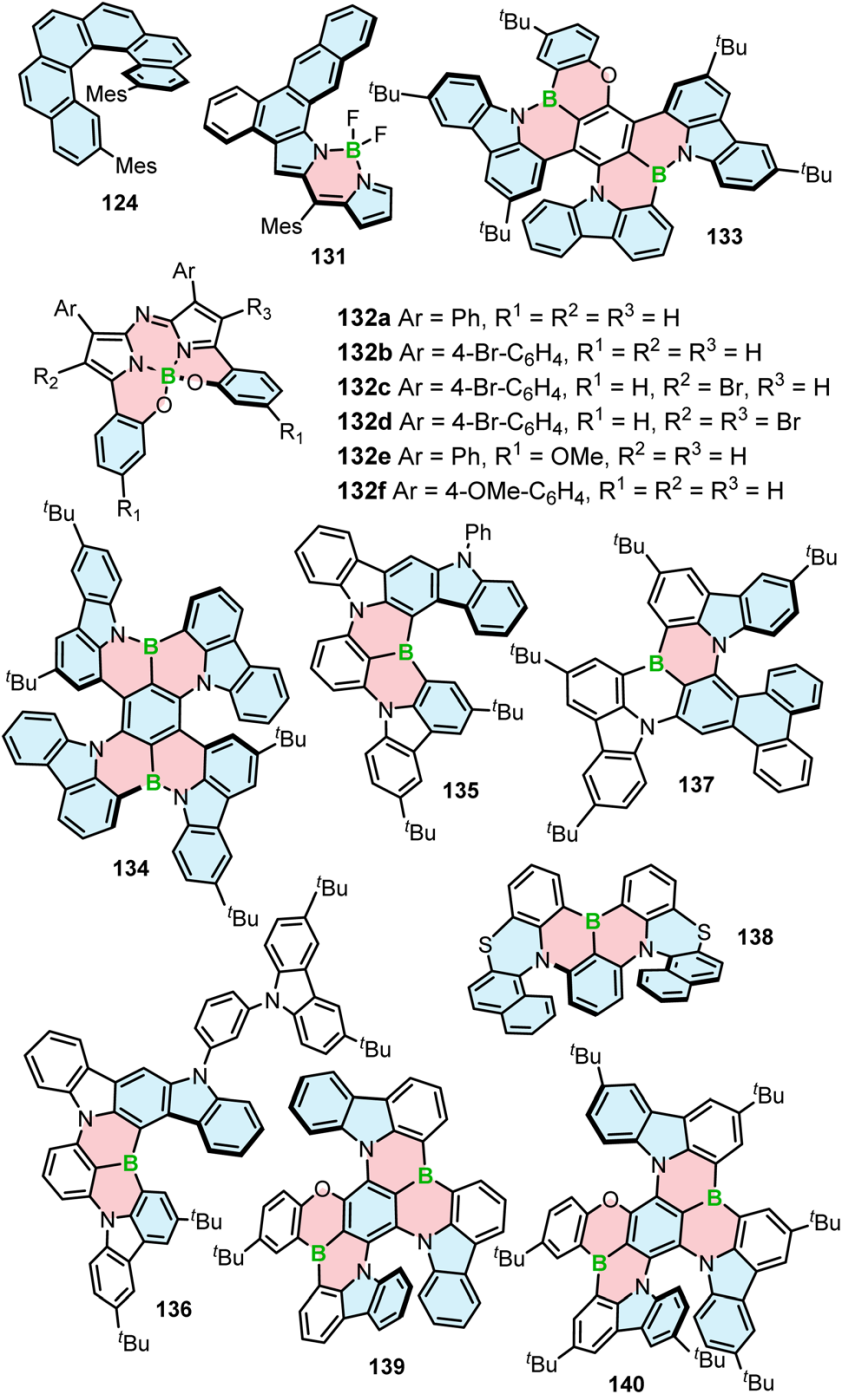
Structures of other compounds discussed in this article.

The introduction of boron into six-membered rings also affects their geometry and therefore, the configurational stability of the corresponding helicenes, although this effect is always combined with the effect of other heteroatoms incorporated in the structure. A simple replacement of one carbon with boron on the inner helicene rim had a rather minor effect with Δ*G*^‡^_exp_ of 103 (*ca.* 35 kcal mol^−1^) and that of pristine carbo[6]helicene essentially equal. When a B=N bond was inserted in 65, the calculated enantiomerization barrier was 26.1 kcal mol^−1^ (PBE0/def2-TZVP), slightly higher than that of the corresponding carbohelicene (24.6 kcal mol^−1^). Nonetheless, the compound slowly racemized at room temperature. Its higher homologue, 66, was considerably more stable and only after heating the sample at 150 °C for 16 h, a barely visible shoulder of the second enantiomer appeared in the corresponding HPLC chromatogram.^82^ The calculated Δ*G*^‡^ for [6]helicene 72 bearing two mesityl substituents was as high as 50.2 kcal mol^−1^. This value could not be confirmed experimentally because of the decomposition of the compound at 200 °C, but without signs of racemization. The authors assign this impressive barrier to the dipolar interaction of the two N–hydrogen atoms on the inner helicene rim in the transition state. While this may contribute to the increase in Δ*G*^‡^, the effect of the Mes substituents cannot be neglected. Substituents at these positions of terminal helicene rings also have impact on their configurational stability, although it is lower than the effect of the groups at the sterically most hindered positions. This becomes clear when Δ*G*^‡^ is compared with structurally similar 124 (Fig. 5). The calculated value for the latter compound is 46.3 kcal mol^−1^, significantly higher than that of carbo[6]helicene lacking these substituents (see above). As mentioned by the authors, this effect is not distinct for [5]helicene 71 (Δ*G*^‡^_exp_ = 25.7 kcal mol^−1^ at 298 K), where the steric clash of these more distant substituents is less pronounced.^83^ The barrier of [6]helicene 59 (Δ*H*^‡^_calc_ = 42.0 kcal mol^−1^, B3LYP/6-31G(d)) is comparable to the barrier of carbo[7]helicene. The enantiomeric excess was retained upon heating the enantiomerically pure sample at 275 °C, which facilitated the fabrication of transistor devices by vacuum deposition.^77^ Double [5]- and [6]helicenes 76a and 76c benefited from the enhancement of Δ*G*^‡^ of a given helicene moiety due to the presence of the second moiety. Accordingly, their Δ*G*^‡^_calc_ was estimated as 33.8 and 49.1 kcal mol^−1^, respectively, and thus were significantly higher than those of pristine carbo[5]- and -[6]helicenes.^86^ It is worth noting that the way in which the helicenes are fused to each other has a tremendous effect on their configurational stability, a feature previously observed for double carbohelicenes.^132^ This positive effect on configurational stability is also clear for double [5]helicene 85. In addition to the rigidification achieved by the formation of C–C bonds (see Scheme 15) and the proper fusion of two helical moieties, the structure is equipped with *t*-Bu groups, which may contribute to its high diastereomerization barrier calculated as 48.1 kcal mol^−1^ (M06-2X^133^-D3/def2-TZVP). The compound did not racemize even when heated to 200 °C in the solid state or 180 °C in *o*-DCB.^89^

The replacement of a benzene ring with a 1,4-oxaborinine ring reduces the configurational stability of a helicene. Δ*H*^‡^ of [6]helicene 35 was 26.6 kcal mol^−1^, indicating that this compound is more stable than pristine carbo[5]helicene but more prone to racemization than carbo[6]helicene.^65^

A relatively low enantiomer-to-*meso*-form diastereomerization barrier of core-unsubstituted double [5]helicene 46a (Δ*G*^‡^_calc_ = 23.0 kcal mol^−1^, B3LYP/6-31G(d)) could be enhanced to 31.8 kcal mol^−1^ upon introduction of the *t*-Bu groups in 46b, though the experimental value was slightly lower (29.0 kcal mol^−1^).^71^ The elongation of each arm by two benzoid units produced double [7]helicene 50 with excellent stability (Δ*G*^‡^_calc_ = 45.1 kcal mol^−1^, B3LYP/6-31G(d)). The high inversion barrier was also confirmed experimentally, as no indication of racemization was observed after heating the sample at 200 °C for 24 h.^74^ As expected, the replacement of four benzoid with thiophene rings significantly lowered the inversion barrier of 56 to Δ*G*^‡^_exp_ of 27.4 kcal mol^−1^ (at 353 K),^76^ again showing a detrimental effect of five-membered rings on configurational stability.

With some exceptions, narrowband boron-containing emitters exhibit quite large chromophores so that the helicene frameworks are part of larger PAH structures consisting of multiple helically chiral units, either fluxional or configurationally stable at room temperature. Consequently, their *P*–*M* interconversion pathways are typically more complex.

Double [7]helicene 41a with Δ*G*^‡^_calc_ (PBE0/def2-TZVP) calculated as 67.5 kcal mol^−1^ exhibits an exceptionally high configurational stability. No racemization was observed, even when the sample was heated in a furnace at 300 °C for 3 h.^69^ The barrier calculated for structurally similar 125 (PBE0/6-311G(d)) was significantly lower (Fig. 4). In the interconversion pathway of this asymmetrical compound, two distinct transition states were found. The first one (Δ*G*^‡^_calc_ = 35.0 kcal mol^−1^) is associated with the inversion of the [6]helicene moiety, while the higher barrier (Δ*G*^‡^_calc_ = 45.1 kcal mol^−1^) – with the inversion of the [7]helicene moiety (Δ*G*^‡^_calc_), present in 41a. Since the inversion of any of these moieties is related with the loss of chiral information, the lower Δ*G*^‡^_calc_ has to be considered in the discussion of its configurational stability.^134^ The barrier of [6]helicene 126 (Fig. 4), calculated at the B3LYP-D3(BJ)/6-31G(d,p) level, is comparable (36.2 kcal mol^−1^), and enabled the fabrication of CP-OLEDs by vacuum deposition.^135^ Interestingly, relatively high configurational stability was achieved for compounds 127–129 consisting of only four angularly fused rings in the helical frameworks (Fig. 4). This is due to the favorable geometry of the thiazine ring and the rigidification of the two phenyl rings by a methylene bridge. Accordingly, Δ*G*^‡^_calc_ (B3LYP/6-31G(d) level of theory in the gas phase) of 127–129 are 28.9, 31.4 and 31.6 kcal mol^−1^, respectively. Much lower inversion barrier for 130 (16.7 kcal mol^−1^) (Fig. 4) shows that replacing oxygen with sulfur in the six-membered rings also enhances Δ*G*^‡^.^136^ Although 127–129 are configurationally stable, these barriers exclude the possibility of thermal evaporation.

As shown in this section, DFT calculations are commonly used to calculate the barriers of the helicenes. It should be noted that these studies should be, where possible, complemented by thermal racemization experiments or tests, in particular when the scaffold is new or equipped with substituents at positions that may affect their Δ*G*^‡^. This is of paramount importance for multiples, where the interconversion pathways are much more complex. Consequently, more than one possible TS may exist for an inversion of a single helicene moiety. Picking the wrong one may lead to erroneous results.

### 3.2. Emission efficiency, luminescence dissymmetry factor and color purity

Materials emitting circularly polarized light have potential for use in a variety of fields, including optical data storage and security,^137–139^ chirality sensing,^140^ probing,^141–143^ and bioimaging.^144^ The most prominent application of CPL emitters is in state-of-the-art CP-OLEDs and 3D displays.^145–148^ CP-OLEDs were proposed to increase the light outcoupling efficiency of OLED displays with anti-glare filters.^145,146^ Conventional OLEDs are equipped with optical elements, such as linear polarizer and a quarter wave plate to eliminate glare from the external light sources. However, when unpolarized light, generated by the OLED, passes through an antiglare filter, around 50% of light is filtered out, hence reducing the display brightness.

Due to their excellent optical properties, chiral boron-containing compounds are well-suited for applications as chiral emitters. Their properties are closely linked to the class they belong to.

In general, the performance of chiral emitters can be evaluated by two important figures-of-merit, that is, luminescence quantum yield and luminescence dissymmetry factor (*g*_lum_). The latter parameter quantifies the degree of circular polarization:1
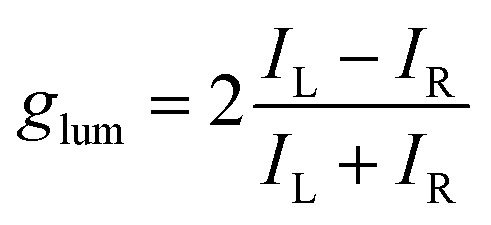
where *I*_L_ and *I*_R_ refer to the intensities of left- and right-handed circularly polarized light. By definition, −2 ≤ *g*_lum_ ≤ 2, while *g*_lum_ = 0 corresponds to non-CP light. The dissymmetry factors typically recorded for CP emitters are in the range of 10^−3^ to 10^−2^, although the values on the order of 10^−2^ are less common.

Alternatively, the dissymmetry factor corresponding to the electronic transition between the emissive excited and ground states can be expressed by the following equation:2
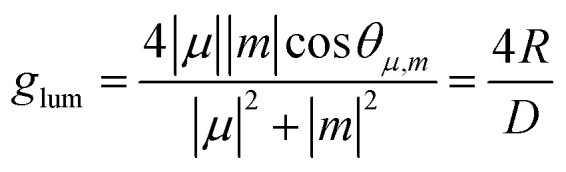
where *R* denotes the rotatory strength, *D* the dipole strength, *m* refers to the magnetic transition dipole moment and *μ* to the electric transition dipole moment for the transition from the excited to the ground states (usually S_1_ → S_0_), while *θ*_*μ*,*m*_ denotes the angle between both vectors. Analogous expressions are valid for *g*_abs_ describing the transition from the thermally equilibrated electronic ground state to the excited state. To maximize the *g*_lum_ value, the vectors should be oriented parallel or antiparallel to one another (cos *θ*_*μ*,*m*_ of 1 or −1) and their magnitudes should be similar. However, *m* is typically small for organic molecules, which contributes to the reduction of this parameter. Since *g*_lum_ is usually several orders of magnitude smaller than the maximum value, it can be considered as the limiting factor in developing high-performing CPL emitters. As other chiral emitters, boron helicenes face the problem of enhancing the |*g*_lum_|, while maintaining high *Φ*_PL_. The common approach is balancing these two relevant parameters, as it is commonly accepted that there is a trade-off between *g*_lum_ and *Φ*_PL_. Indeed, compounds exhibiting large *g*_lum_ tend to have low *Φ*_PL_ and *vice versa*, although the studies by Mori indicate that |*g*_lum_| can be enhanced independently from *Φ*_PL_.^149^ In principle, to enhance |*g*_lum_|, in addition to optimizing angle *θ*_*μ*,*m*_, the magnitude of vector |*μ*| should be reduced, which however may have a negative impact on *Φ*_PL_. Therefore, a better approach is to increase the magnitude of vector |*m*| relative to |*μ*|. For a detailed discussion of the relationship between *μ* and *m*, readers are directed to the original papers and reviews on the topic.^149,150^

To evaluate the performance of chiral emitters and enable the comparison between the compounds belonging to different classes, two parameters have been introduced for samples in solution combining *Φ*_PL_ and |*g*_lum_|. The first one, circular polarization luminosity *Λ*_CPL_, proposed by Nagata and Mori, is described by eqn (3):3
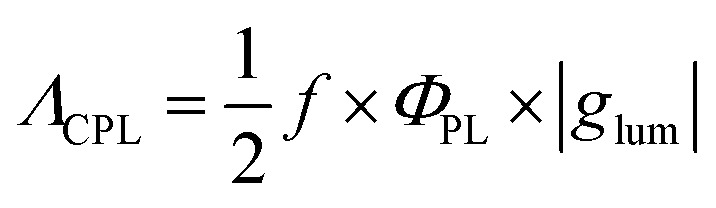
where *f* is the oscillator strength corresponding to the transition from S_0_ to the excited state. The second measure, CPL brightness *B*_CPL_ (eqn (4)), was introduces by Arrico, Di Bari and Zinna.^9^4
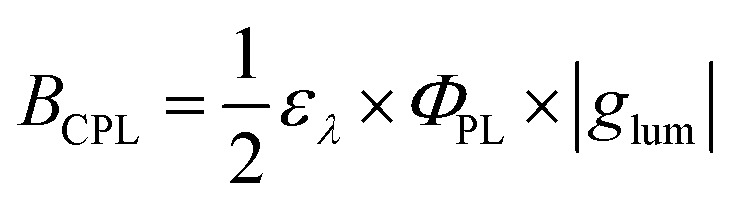
Here, *ε*_*λ*_ corresponds to the molar absorption coefficient at the excitation wavelength. Thus, both parameters take into account the absorption efficiency. In the following discussion, we focus mainly on *Φ*_PL_ and |*g*_lum_|. Table 1 summarizes the optical properties of the compounds discussed in this article, including these parameters in addition to full width at half maximum (FWHM), and fluorescence lifetimes (*τ*_FL_).

**Table 1:** Emissive properties of boron-containing helicenes in solution

Cpd	Solvent^a^	*λ*_em_ b [nm]	*Φ*_PL_ c	FWHM^d^	*τ*_FL_ e [ns]	|*g*_lum_| × 10^−3^^f^	Ref.
5	CH_2_Cl_2_	435	0.069	—	5.3	0.7	54
6	CH_2_Cl_2_	404	0.21	—	4.1	0.9	54
7	CH_2_Cl_2_	443	0.074	—	5.5	1	54
8	CH_2_Cl_2_	427	0.49	—	3.2	2.3	54
13a	CH_2_Cl_2_	459	0.20	—	2.44	0.68	55 and 57
13b	CH_2_Cl_2_	462	0.18	—	—	—	55
13c	CH_2_Cl_2_	477	0.24	—	2.87	1.2	55 and 57
14a	CH_2_Cl_2_	443	0.43	—	—	—	55
14c	CH_2_Cl_2_	446	0.47	—	—	—	55
15a	CH_2_Cl_2_	433	0.15	—	2.56	1.7	57
15b	CH_2_Cl_2_	449	0.14	—	3.02	3.2	57
16a	CH_2_Cl_2_	436	0.15	—	1.87	1.2	57
16b	CH_2_Cl_2_	450	0.12	—	2.50	1.7	57
17	CH_2_Cl_2_	448	0.18	—	—	—	56
18	CH_2_Cl_2_	515	0.28	—	—	1.6	56
19a	CH_2_Cl_2_	510	0.31	—	—	2.2	56
25a	Toluene	495	0.29	—	4.8	0.25	60
	MeCN	499	0.28	—	6.4	—	
25b	Toluene	502	0.30	—	7.7	0.95	60
	MeCN	510	0.29	—	9.9	—	
25c	Toluene	586	0.13	—	19.9	3.5	60
	MeCN	*ca.* 680	<0.005	—	—	—	
30	CH_2_Cl_2_ toluene	469	0.17	—	—	0.9	63
		465	0.14	—	—	—	
40	Toluene	615	0.89	21 (0.07 eV)	6.1 (p), 89 μs (d)	—	68
41a	Toluene	660	1.00	—	13.0 (p), 16.4 μs (d)	*ca.* 2 (CH_2_Cl_2_)^g^	69
	Toluene	662	1.00	38	12.0 (p), 16.6 μs (d)	—	70
41b	Toluene	684	0.99	—	12.2 (p), 43.9 μs (d)	<2 (CH_2_Cl_2_)^g^	69
	Toluene	692	1.00	38	14.2 (p), 46.4 μs (d)	—	70
41c	Toluene	696	0.90	—	11.0 (p), 26.8 μs (d)	*ca.* 1.2 (CH_2_Cl_2_)^g^	69
46a	CH_2_Cl_2_	430	0.68	—	3.75	—	71
	CH_2_Cl_2_	434	0.61				73
46b	CH_2_Cl_2_	436	0.65	—	3.96	1.7	71
46c	CH_2_Cl_2_	441	0.52	—	—	—	73
49	CH_2_Cl_2_	490	0.03	—	—	—	72
50	CH_2_Cl_2_	487	0.26	—	—	—	74
52a	CH_2_Cl_2_	653	0.20	—	2.84	—	75
52b	CH_2_Cl_2_	623	0.29	—	4.52	—	75
56	Cyclohexane	392	0.06	—	—	—	76
59	CH_2_Cl_2_	447	—	—	—	—	77
65	CH_2_Cl_2_	404, 422 (m)	0.30	—	—	—	82
66	CH_2_Cl_2_	419, 436 (m)	0.21	—	—	—	82
67	CH_2_Cl_2_	395, 411 (m)	0.42	—	—	—	82
71	CH_2_Cl_2_	407, 428 (m)	0.10	—	4.7	4.2	83
72	CH_2_Cl_2_	434, 459	0.17	—	7.1	13.3	83
76a	CH_2_Cl_2_	505	0.83	—	6.4	—	86
76b	CH_2_Cl_2_	504	0.70	—	6.7	—	87
76c	CH_2_Cl_2_	528	0.80	—	7.1	0.75	86
76d	CH_2_Cl_2_	526	0.64	—	7.4	1.1 (THF)	87
76e	CH_2_Cl_2_	505	0.67	—	5.3	—	87
80	THF	384	0.63	—	—	—	88
81	THF	431	0.05	—	—	—	88
83	THF	565	0.39	—	—	—	88
84	Toluene	524	0.99	24	6.5	—	89
85	Toluene	522	0.65	22	4.0	1.0	89
87	Cyclohexane	485	0.81	—	—	—	90
	Benzene	493	0.75	—	—	—	
	CHCl_3_	500	0.63	—	—	—	
	Toluene	491	0.90	—	6.31	—	92
93	Toluene	516	0.79	—	5.28	—	92
90	Cyclohexane	472	0.69	—	—	—	91
	Benzene	484	0.65	—	—	—	
	CH_2_Cl_2_	484	0.66	—	—	—	
97a	Toluene	512	0.24	—	—	—	94
99	Toluene	592	0.23	—	—	—	94
100	Toluene	410	0.19	—	—	—	94
101	Toluene	569	0.49	—	—	—	95
102	Toluene	623	0.34	—	—	—	95
103	CHCl_3_	587	0.77	—	10.1	1.4	44
104	CH_2_Cl_2_	—	0.33	—	2.81	—	105
105a	CH_2_Cl_2_	521	0.05	—	1.86	—	106
105b	CH_2_Cl_2_	563	0.12	—	6.78	—	106
105c	CH_2_Cl_2_	579	0.05	—	2.40	—	106
105d	CH_2_Cl_2_	621	0.08	—	7.87	—	106
107a	CH_2_Cl_2_	625	0.59	—	5.33	0.03	107
107b	CH_2_Cl_2_	649	0.56	—	7.17	—	107
107c	CH_2_Cl_2_	640	0.31	—	3.39	—	107
107d	CH_2_Cl_2_	668	0.12	—	1.60	—	107
108a	CH_2_Cl_2_	682	0.30	—	4.22	1.29	107
108b	CH_2_Cl_2_	708	0.24	—	4.28	1.30	107
108c	CH_2_Cl_2_	695	0.16	—	2.88	1.22	107
108d	CH_2_Cl_2_	719	0.10	—	2.07	—	107
(*S*)-110	CHCl_3_	*ca.* 570	0.44	—	—	0.85	109
(*R*)-110	CHCl_3_	*ca.* 570	0.46	—	—	0.71	109
115a	CH_3_CN	623	0.65, 0.73 (CH_2_Cl_2_)	—	—	4.7	110
115b	CH_3_CN	635	0.73, 0.92 (CH_2_Cl_2_)	—	—	3.3	110
115c	CH_3_CN	637 (ref. 110)	0.52 (ref. 110)	—	11.3	4.3	110 and 112
		643 (ref. 112)	073 (ref. 112)				
115d	CH_3_CN	675	0.28	—	—	4.2	110
115e	ACN	636	0.48	—	8.3	—	112
116	CH_2_Cl_2_	665	0.02	—	—	—	111
117	CH_2_Cl_2_	741	0.24	—	—	1.4	111
118	CH_2_Cl_2_	751	0.14	—	—	5.7	111
122	CH_2_Cl_2_	615	0.56	—	—	—	115
	Hexane	622	0.49	—	—	3.7	
125	Toluene	617	0.96	38 (0.13 eV)	—	1.41	134
126	Toluene	527	0.9	35	—	—	135
127	Toluene	511	0.72	46 (0.216 eV)	4.83 (p), 16.2 μs (d)	—	136
128	Toluene	500	0.88	43 (0.207 eV)	5.1 (p), 8.2 μs (d)	1.0–2.0	136
129	Toluene	497	0.87	44 (0.216 eV)	5.1 (p), 23.1 μs (d)	1.0–2.0	136
131	CH_2_Cl_2_	—	0.53	—	3.55	—	105
132a	Toluene	746	0.51 (1% in pyridine)	36	—	—	113
132b	Toluene	760	0.46 (1% in pyridine)	36	—	—	113
132c	Toluene	769	0.35 (1% in pyridine)	38	—	—	113
132d	Toluene	776	0.36 (1% in pyridine)	36	—	—	113
132e	CHCl_3_	782	0.18	40	—	—	113
132f	CHCl_3_	742	0.17	36	—	—	113
133	Toluene	522	0.90	28 (0.13 eV)	4.7	—	159
134	Toluene	547	0.86	26 (0.11 eV)	4.0	—	159
135	Toluene	521	0.99	21 (0.09 eV)	6.8 (p), 239 μs (d)	—	160
136	Toluene	520	0.98	22 (0.09 eV)	7.1 (p), 160 μs (d)	—	160
137	Toluene	523	0.96	34	—	—	161
138	Toluene	520	0.98	46	—	2.1	162

a When a different solvent was used for a particular measurement, it is given in parentheses.

b Emission maximum.

c Photoluminescence quantum yield.

d Full width at half maximum.

e (Amplitude-weighted average) fluorescence lifetime.

f Luminescence dissymmetry factor.

g The value estimated from the *g*_lum_ plot. The exact value was not given. m – main band, p – prompt component, d – delayed component.

The fluorescence quantum yields of non-extended carbohelicenes are extremely low, ranging from *ca.* 0.04 to 0.02 (in dioxane) for carbo[5]-, -[6]-, and -[7]helicenes and further decreasing with the elongation of the helix.^151,152^ This is due to the pronounced intersystem crossing (ISC) in these archetypal helicenes.^152,153^ In addition, these compounds display weak absorption. For instance, *ε* for the lowest-energy absorption bands of carbo[5]helicene at 393 nm is 200 m^−1^ cm^−1^. This is due to the symmetry-forbidden character of the corresponding S_0_ → S_1_ transition.^154^ The emission efficiency of helically chiral PAHs can be enhanced through doping with heteroatoms, with boron occupying a prominent position. The introduction of boron atoms, usually in combination with other heteroatoms, into helical scaffolds has a tremendous impact on their emissive properties. In a collaborative work with the group of Michael Ingleson, we showed that the replacement of a single benzoid ring in [6]helicene 66 with an azaborine ring enhanced *Φ*_FL_ to 0.21 in CH_2_Cl_2_, while maintaining the *g*_abs_ value. Accordingly, |*g*_abs_| at 369 nm is 1.8 × 10^−3^, comparable to that of all-carbon derivative.^82^ The emission bands of shorter homologue 65 and helicene 67 are hypsochromically shifted *vs.* that of 66 (404 and 395 nm *vs.* 419 nm for the weaker 0–0 transitions). These derivatives display *Φ*_FL_ of 0.30 and 0.42. Stronger emission in the latter case is attributed to the presence of a cyclopendadiene ring, which tends to enhance *Φ*_FL_.^155,156^ The enhancement of both *Φ*_FL_ and *ε* is attributed to the altering of the symmetry of the frontier orbitals relative to archetypal carbohelicenes, leading to higher *f* due to the allowed S_1_ → S_0_ transition. This is also the reason for the increased emission efficiency of 71 and 72 with two embedded azaborine rings (*Φ*_FL_ of 0.10 and 0.17, CH_2_Cl_2_), associated with the shorter fluorescence lifetimes *τ*_FL_ compared to the structurally related carbohelicenes bearing Mes substituents (*e.g.*124, Fig. 5).^83^ One of the derivatives, BN-[6]helicene 72, exhibits exceptionally intensive CPL with |*g*_lum_| of 1.33 × 10^−2^ (CH_2_Cl_2_), while |*g*_lum_| for pristine carbo[6]helicene is low, only 0.9 × 10^−3^ (CH_2_Cl_2_).^157^ The value of 72 is also *ca.* three times higher than that of 71 (4.2 × 10^−3^). According to TD-DFT calculations at the B3LYP/cc-pVDZ,^158^ this is likely due to a more favorable orientation of the *m* and *μ* vectors for 72 than for 71 (135° *vs.* 112°). These two works highlight the impact of a B=N bond, isoelectronic to a C=C bond, on the electronic and optical properties of the helical systems.

Likewise, azaborole helicenes exhibit appealing emissive properties in solution and in the solid state, which can be modulated by changing the helical framework and the boron substituents. *Φ*_FL_ of 13a–c reaches moderate values of 0.24 and 0.47 for 14a–c. The emission maxima of azabora[7]helicenes 13a–c in CH_2_Cl_2_ are positioned between 459 and 477 nm and correspond to the blue or blue–green emission color, whereas *λ*_em_ of double helicenes 14a–c are hypsochromically shifted (443–446 nm). Importantly, high emission intensity could be retained in the solid state with the highest *Φ*_PL_ of 0.23 and 0.25 for phenyl derivatives 13c and 14c, respectively. The emission color of azabora[7]helicenes changed upon going from solution to the solid state from blue to green for alkyl derivatives and blue–green to yellow for 13c.^55^ However, |*g*_lum_| of 13a,c are quite low (6.8 × 10^−4^ and 1.2 × 10^−3^, respectively). Higher values were achieved for singly truncated 15a,b and 16a,b with the highest |*g*_lum_| for 15b of 3.2 × 10^−3^, respectively, though the compounds showed somewhat lower *Φ*_FL_ in CH_2_Cl_2_ solution in the range of 0.12–0.15. Remarkably, *Φ*_PL_ in the solid state reached the values as high as 0.53 and 0.51 for 15a and 15b, respectively. These are the highest values reported for boron-containing helicenes and are highly exceptional for carbo- and heterohelicenes in general. In a glassy matrix (2-methyltetrahydrofuran) at 79 K, these azaborole helicenes show another set of emission bands ascribed to phosphorescence and corresponding to the averaged lifetimes of 0.18–0.54 s.^57^

**Fig. 6:**
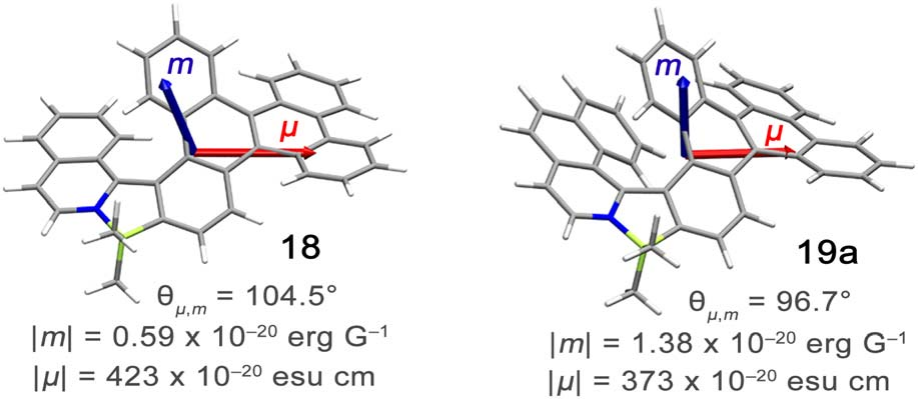
DFT-optimized structures of 18 and 19a and the spatial arrangement of the electric (*μ*, red) and magnetic (*m*, blue) dipole moments in the S1 excited state at the TD-M06-2X/def2-SVP level.^56^

We could enhance the emission properties of 13a by lateral extension. Thus, 19a exhibits *Φ*_FL_ of 0.31 in CH_2_Cl_2_, 50% higher than that of parent molecule 13a. In addition, the fusion of the additional phenanthrene unit shifted *λ*_em_ bathochromically to 510 nm. Consequently, the compound shows green emission color. Likewise, *Φ*_FL_ of laterally extended [6]helicene 18 was high (0.28), while that of [5]helicene 17 significantly lower (0.18). Not only did the lateral extension improve *Φ*_FL_, but it also enhanced |*g*_lum_|. Accordingly, the |*g*_lum_| value determined for (*M*)-19a was 2.2 × 10^−3^ (at 501 nm, CH_2_Cl_2_), *ca.* three times higher than that of the methyl derivative of (*P*)-13a and reflect the higher oscillator strength of the lowest-energy absorption band of 19a. In addition, the |*g*_lum_| value of this compound was higher when compared to that of its shorter homologue 18 (1.6 × 10^−3^ at 507 nm). Our TD-DFT calculations at the M06-2X/def2-SVP level satisfactorily reproduced the trend and the signs of the experimentally determined *g*_lum_. The analysis of the *m*, *μ*, and *θ*_*μ*,*m*_ implied that the nearly 2.5-fold increase in *m* for 19a compared to its shorter homologue is likely responsible for the enhanced *g*_lum_ value of this compound, even though the angle between both vectors (96.7° for 19a*vs.* 104.5° for 18) was less favorable (Fig. 6).^56^ In the set of azaborahelicenes published by Crassous and colleagues, the highest *Φ*_FL_ were recorded for [6]helicenes. While *Φ*_FL_ of 6 was comparable to that of [7]helicene 13a (0.21 *vs.* 0.20, respectively), it could be enhanced to 0.49 for 8 with two azaborole rings. The helical extension dramatically reduced the emission efficiency of 5 and 7 to *ca.* 0.07. This could indicate that the behavior of carbo[6]helicene to which azaborole rings are fused either on one or both sides, dominates the properties of these compounds. In addition, 5–7 show relatively low |*g*_lum_| (≤1 × 10^−3^). The highest value of 2.3 × 10^−3^ was reported for 8. Despite the good optical properties of this [6]helicene, its configurational stability (see above) is too low for practical applications.^54^ It also holds true for azabora[5]helicenes 25a,b with the aryl substituents at a sterically hindered position. Their *Φ*_FL_ values in toluene and MeCN are *ca.* 0.30, but the |*g*_lum_| are very low, below ≤1 × 10^−3^. This could be enhanced for amino derivative 25c, but at the expense of its emission efficiency, which is reduced to 0.13 in toluene and below 0.01 in a more polar solvent.^60^

Even though the incorporation of thiophene lowers the emission efficiency of helicenes, *Φ*_FL_ of azaborathia[9]helicene 30 reaches the values of 0.14 in toluene and 0.17 in CH_2_Cl_2_, the highest for thiophene-containing non-extended helicenes. This demonstrates the beneficial effect of incorporating a boracycle. The emission wavelength of this blue emitter is positioned at 465 and 469 nm, respectively, which falls in the optimal range for blue emitters for OLEDs. However, the |*g*_lum_| value of 30 is relatively low (*ca.* 0.9 × 10^−3^, CH_2_Cl_2_).^63^ In contrast, *Φ*_FL_ of blue-emissive BO-helicene 56 is 0.06 in cycloxexane (*λ*_em_ = 392 nm) and 0.04 in THF (*λ*_em_ = 411 nm), implying that the B–O motif does not have such an impact on the electronic spectra of thiohelicenes.^76^ On the other hand, the effect of B–O motifs is evident in OBO-derivatives. Double [5]helicenes 46a–c exhibit strong emission with *Φ*_FL_ in the range of 0.52–0.68 in CH_2_Cl_2_.^71,73^ The helical elongation results in reducing the fluorescence efficiency of double [7]helicene 50 to 0.26 and a bathochromic shift of the emission from *λ*_em_ of 430–441 nm for 46a–c to 487 nm for 50, showing the green–yellow emission color.^74^ Interestingly, 46a and 46b are also highly emissive in the solid state (*Φ*_PL_ of 0.46 and 0.26, respectively). The latter compound, configurationally stable due to the presence of *t*-Bu groups, displays a moderate |*g*_lum_| value of 1.7 × 10^−3^, CH_2_Cl_2_.^71^ The replacement of a covalent B–C with a dative B–N dative bond in 52a and 52b leads to a considerable red-shift of the emission maxima to 653 and 623 nm (red emitters) and decrease in their *Φ*_FL_ to 0.20 and 0.29, depending on the ligand on boron (Ph *vs.* F), when compared to 46a.^75^

Helicenes with a N–B–N motif generally exhibit strong emission. For instance, *Φ*_FL_ of helicenes 76a,c reach 0.80–0.83 in CH_2_Cl_2_, whereas those of NH derivatives 76b,d,e are somewhat lower (0.64–0.70). Their *λ*_em_ are centered at 504–528 nm, corresponding to green or green-yellow emission color. On the other hand, the |*g*_lum_| of 76c–e is low (7.5 × 10^−4^–1.1 × 10^−3^) and could not be determined for some derivatives.^86,87^ The *Φ*_FL_ of double helicene 84 with two N–B–N close to unity in toluene is reduced to 0.65 for 85 upon rigidification. The formation of the additional two C–C bods essentially does not affect the position of the emission maxima (522–524 nm). Even though FWHM are only 24 and 22 nm, respectively, both compounds show pronounced vibronic progressions, which contribute to the spectra broadening. Moreover, the |*g*_lum_| is quite low, only 1.0 × 10^−3^.^89^

BODIPY helicenes and their analogues exhibit generally strong absorption in the visible spectral region. For instance, the lowest-energy absorption bands of helically chiral derivatives, discussed in Section 2.5, are positioned between 495 and 691 nm. Their emission bands are in the range of 521–751 nm with low to very good *Φ*_FL_ from 0.02 for 116 (CH_2_Cl_2_)^111^ to 0.92 for 115b (CH_2_Cl_2_),^110^ depending on the molecular design and the targeted application. Their optical properties have been modulated by substituent effects and doping with heteroatom. As shown for highly photostable BODIPYs 107a–d and 108a–d, introducing (4-(*tert*-butyl)phenyl)thio peripheral groups contributed to a red-shift of absorption and emission of these compounds compared to those bearing 4-(*tert*-butyl)phenyl groups. The additional replacement of the fluoride with Ph substituents on boron produced near-infrared (NIR) emitter 108d with *λ*_em_ of 719 nm (CH_2_Cl_2_). In this set of compounds, the combination of thiol and Ph groups along with the helical extension leads to a decrease in the emission efficiency to reach the value of 0.10 for 108d. The other extremum is 107a with *λ*_em_ of 625 nm and *Φ*_FL_ of 0.59 (CH_2_Cl_2_).^107^

While other boron-containing helicenes, are usually designed to exhibit strong emission, BODIPY derivatives were identified as promising triplet photosensitizers. A prerequisite for this application is a high quantum yield of ISC (*Φ*_ISC_). As reported by Sapir and Vander Donckt, the ISC rates, *k*_ISC_, increase from carbo[4]- to -[8]helicene.^153^ This trend is also visible in the well-established class of BODIPY dyes. For instance, 104 exhibits *Φ*_FL_ of 0.33 in CH_2_Cl_2_, while its less twisted regioisomer 131 (Fig. 5) *Φ*_FL_ of 0.53 corresponding to the *Φ*_ISC_ of 0.63 and 0.23, and *k*_ISC_ 2.24 × 10^8^ s^−1^ and 6.48 × 10^7^ s^−1^, respectively. Thus, the *Φ*_ISC_ value of 131 is nearly three times lower than that of more twisted 104.^105^ This tendency of helicenes to undergo ISC is desirable for applications as heavy-atom-free triplet photosensitizers.^163^ In addition to high *Φ*_ISC_, such compounds should possess significant absorption in the visible or near-UV region. Here, boron derivatives outperform carbohelicenes, as the latter display weak lowest-energy absorption bands. On the other hand, ISC in boron-containing helicenes is usually less efficient.

To enhance ISC in 105a–d, one of the pyrrole rings of a classical BODIPY was replaced with thiazole.^106^ The substitution of this ring with the cyano group to produce a push–pull system and placing the Ph substituents on boron shifted the absorption and emission bathochromically to reach *λ*_em_ of 621 nm for BODIPY 105d*versus* 521 for 105a (CH_2_Cl_2_). This rational engineering of a BODIPY chromophore resulted in compounds with high triplet conversion, confirmed by the singlet oxygen (^1^O_2_) generation experiments (singlet oxygen quantum yields, *Φ*_Δ_ of 0.28–0.58 in EtOH) and transient absorption spectroscopy. This feature allowed some of these helicenes to be successfully tested for photodynamic therapy but is also responsible for their low *Φ*_FL_ (0.05–0.12, CH_2_Cl_2_). BODIPYs 115c and 115e also proved to generate ^1^O_2_ with *Φ*_Δ_ of 0.12 and 0.40, respectively.^112^ The ISC process for 115c was inefficient due to the large singlet–triplet energy gap (Δ*E*_ST_) and thus, the compound was highly fluorescent (*Φ*_FL_ = 0.73, MeCN)^112^ despite the twisted structure. Noteworthy, this type of chiral BODIPYs usually exhibit intensive emission in the red region of the electromagnetic spectrum, which distinguishes them from other helically chiral BODIPY dyes. For instance, *Φ*_FL_ of 115a and 115b were recorded in CH_2_Cl_2_ as 0.73 and 0.92.^110^ Even in the NIR region, the emission remains strong, as reported for 117 and 118 (0.24 and 0.14 at *λ*_em_ of 741 and 751 nm, respectively).^111^ The high value of *Φ*_Δ_ for 115e was realized by the introduction of an anthracene substituent in the *meso*-position. This contributes to the enhancement of the ISC *via* the intermediate T_2_ state (lying below the S_1_ state), associated with the anthryl moiety.^112^

The chiroptical properties of BODIPYs are characterized mainly for these *O*-BODIPYs. The *g*_lum_ values determined for (*R*)-110 and (*S*)-110 are quite low, 0.71 × 10^−3^ and −0.85 × 10^−3^ in CHCl_3_, respectively.^109^ Much higher |*g*_lum_|, in the range of 3.3 × 10^−3^–4.7 × 10^−3^ in MeCN were recorded for *O*-BODIPYs 115a–d, with the highest value for 115a, which also displays strong emission.^110^*N*,*N*,*O*,*C*-BODIPY 122 exhibits the |*g*_lum_| value in hexane (3.7 × 10^−3^) comparable to those of 115a–d. A higher |*g*_lum_| was recorded for helically extended 118 (5.7 × 10^−3^. CH_2_Cl_2_), but this value was associated with low *Φ*_FL_ (*vide supra*).^111^ BODIPY analogues 107 and 108 display a weaker CPL signal with |*g*_lum_| of up to 1.3 × 10^−3^ for 108b. Although not all derivatives could be resolved by HPLC and characterized, it appears that the values for the helically extended 108 are much higher than for 107.^107^

The absorption and emission spectra of BODIPYs can be red-shifted upon substitution of the *meso* carbon atom with nitrogen to produce aza-BODIPYs 132a–f. These compounds show moderate to high *Φ*_FL_ of 0.17–0.51 in the NIR region (*λ*_em_ of 742–782 nm). Similar to the *B*,*O*-chelated dipyrromethanes,^164^ the rigidification of the molecular structures through boron gave rise to narrowband emission with FWHM in the range of 36–40 nm.^113,114^

In addition to conventional fluorescent emitters and their characteristics, several years ago, a conceptually new approach was introduced to designing emitters that exhibit narrowband emission.^66^ This approach, based on multi-resonance effect, has been intensively exploited to develop materials combining the MR characteristics with thermally activated delayed fluorescence (TADF), which ensures high *Φ*_FL_. The latter process, relying on a thermally promoted reverse ISC (RISC), enables triplet exciton harvesting *via* upconversion of triplet into singlet excitons, which requires a small Δ*E*_ST_, usually below 0.2 eV. Theoretically, this allows to enhance the internal quantum efficiency (IQE) in OLEDs from up to 25% (determined by the ratio of electrically generated singlet and triplet excitons of 1 : 3) to *ca.* 100%. Traditional TADF emitters consist of weakly coupled donor (D) and acceptor (A) moieties with spatially separated HOMOs and LUMOs. These compounds show charge-transfer (CT) characteristics of S_1_, which, combined with large structural relaxation in the excited state, results in broad emission bands and reduced radiative deexcitation.

**Fig. 7:**
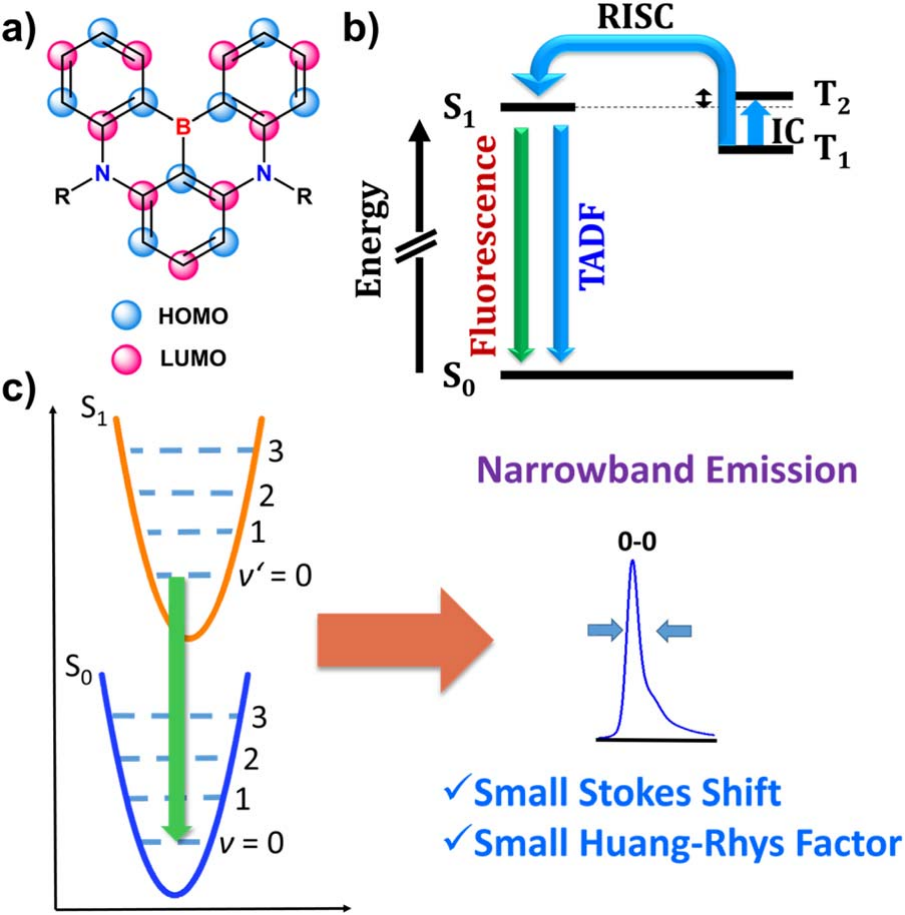
(a) Frontier molecular orbitals in MR-TADF frameworks. (b) Predicted reverse intersystem crossing pathways present in MR-TADF scaffolds. (c) Correlation between the vibronic coupling and emission spectrum.

Another reason for the broadening of emission spectra is vibronic coupling. The relative intensity of the 0–1 vibronic line (from *ν* = 0 of the singlet excited state (S_1_) to *ν* = 1 of the ground state (S_0_)) and zero-phonon line (between the *ν* = 0 vibrational states of S_0_ and S_1_) is determined by the Huang–Rhys factor (*S*), a dimensionless factor which is a measure of the displacement between the potential energy surfaces (PESs) of S_0_ and S_1_.^165^ The increase in *S*, which is correlated with more pronounced changes between S_0_ and S_1_, leads to a vibrational fine structure and hence the broadening of the emission spectrum. Therefore, the Huang–Rhys factor should be as small as possible. If the S_1_ and S_0_ equilibrium geometries are similar, the 0–0 vibronic transition prevails to produce narrowband emission,^166^ the feature commonly observed in MR-TADF materials (Fig. 7c). These materials are based on highly rigid molecular frameworks that typically use alternating boron and nitrogen (or oxygen) atoms to create an opposite resonance effect (Fig. 7a). In contrast to traditional TADF emitters, the HOMO and LUMO in multi-resonant structures are localized on different atoms, resulting in the reduced Δ*E*_ST_ and weak vibronic coupling.

A narrow emission band translates directly to the emission color purity, an important characteristics of emissive functional materials. To meet the increasing demand for monochromatic red (R), green (G), and blue (B) colors, the International Telecommunication Union (ITU) announced in 2012 a new color gamut standard for ultra-high-definition televisions (UHDTVs) called the Broadcast Service Television 2020 (BT.2020). This color gamut includes the primary colors with Commission Internationale de l’Éclairage (CIE) coordinates for R, G, and B of (0.708, 0.292), (0.170, 0.797), and (0.131, 0.046), respectively.^167^ The design and synthesis of emissive materials for OLEDs should aim to achieve narrow emission bands that fall within this coordinate range, leading to displays with high color purity. This parameter should be ideally combined with high emission efficiency and large dissymmetry factors.

The pioneering work of Hatakeyama, demonstrating the MR effect for compound 38 (DABNA-1) (see Scheme 7) with arylamine moieties^66^ have sparked a massive interest in the search for boron-based MR-TADF materials with improved characteristics. While a number of achiral compounds have been synthesized and investigated to date, chiral derivatives are rare in this field. The few MR-TADF helicenes manifest narrowband emission with high luminescence efficiency. The opposite resonance effect of B/N atoms, the reduced vibronic coupling and vibrational relaxation in these compounds result in emission peaks with an extremely small FWHM.

However, MR-TADF compounds often show relatively high Δ*E*_ST_ values compared to the conventional D–A intramolecular CT (ICT)-type TADF materials, larger than the thermal energy at 300 K (*k*_B_*T* ≈ 25.9 meV). This results in the limited rate of triplet harvesting (*k*_RISC_ ≈ 10^4^–10^5^ s^−1^) contributing to a significant efficiency roll-off of devices. Thus, the RISC acceleration would be essential to alleviate this problem. A rational approach would be to decrease the Δ*E*_ST_. This could be realized by enhancing the ICT character, but the downside is broadening of emission spectra and energy losses. However, in properly designed compounds with close lying T_1_ and higher triplet states, the RISC mechanism can involve, *e.g.* T_2_ state. Indeed, it was shown for some MR-TADF emitters that the mechanism can proceed *via* three steps: reverse internal conversion (RIC) from T_1_ to T_2_ (or higher states), followed by RISC from T_2_ to S_1_, and subsequent fluorescence from S_1_ to S_0_ (Fig. 7b).^168–170^ Thus, minimizing the Δ*E*_S_1_T_2__ should also be considered in the design of boron-based MR-TADF emitters.

In 2020, Yasuda proposed a new design paradigm for MR-TADF emitters, extending the frontier molecular orbital (FMO) strategy to larger PAHs by arranging the N and B atoms in the *para*-N-π-N and *para*-B-π-B configuration around the central benzene ring.^68^ This strengthens the relative electron-donating and -withdrawing ability of the donor and acceptor, respectively, leading to the bathochromically shifted absorption and emission spectra. Consequently, 40 exhibited red emission with *λ*_em_ at 615 nm. Importantly, this compound realized very narrow emission with FWHM of 21 nm (0.07 eV) owing to the rigid molecular framework, strong emission (*Φ*_PL_ = 0.89) in toluene reasonable TADF characteristics, although *k*_RISC_ was not very high (1.2 × 10^4^ s^−1^) due to the fact that Δ*E*_ST_ values is still too high (0.19 eV).

This design strategy was used later to construct double [7]helicenes 41a–c.^69,70^ Their structures, consisting of four carbazole units, comprised two *para*-N-π-N and one *para*-B-π-B motifs resulting in deep-red emission with the fluorescence bands positioned between *ca.* 660 nm for the non-substituted derivative and 696 nm for 41c in toluene, without compromising the MR feature. Excellent *Φ*_PL_ of 0.90–1.00 could be realized due to the TADF characteristics enabled by the sufficiently small Δ*E*_ST_ of 0.18–0.22 eV in toluene^69^ (0.16–0.18 eV for 41a,b in toluene),^70^ although, like in the case of 40, their RISC is relatively slow (*k*_RISC_ ≈ 10^4^ s^−1^). The rigid structures of these double helicenes resulted in small Stokes shifts and FWHM of 38 nm in toluene.^70^ In contrast to 40, their CPL properties have been investigated revealing moderate |*g*_lum_| of up to 2 × 10^−3^ in CH_2_Cl_2_.

Stitching either one or two carbazole moieties to the central ring in a different way than in 41b produced regioisomeric 125 (one carbazole moiety shifted, Fig. 4)^134^ and other structurally similar compounds 133, and 134 (two moieties shifted, Fig. 5)^159^ with one or two B–N covalent bonds, respectively. This structural modification also converts a [7]helicene into a [6]helicene unit. The formation of the bond with nitrogen reduces the electron-withdrawing ability of boron and hence, shifts the emission hypsochromically from *λ*_em_ positioned at 692 nm (toluene) for 41c^70^ to 617 nm for 125 with one C–B bond^134^ and 522 and 547 for 133 and 134 with two C–B bonds.^159^ These *λ*_em_ correspond to red (125 and 134) or yellow (133) emission colors. Their high *Φ*_PL_ can be attributed to the large *f* values and highly rigid π-conjugated frameworks. Accordingly, 125 shows strong emission with *Φ*_PL_ as high as 0.96 in degassed toluene and small FWHM of only 38 nm (0.13 eV). *Φ*_PL_ of 133 and 134 values, although somewhat lower (0.90 and 0.0.86 in degassed toluene), can be essentially retained in doped films. Their small FWHM of 28 nm (0.13 ev) and 26 nm (0.11 eV), respectively, indicate high color purity. However, due to quite large Δ*E*_ST_ values (0.36–0.40 eV), these compounds do not show TADF characteristics. In addition, these materials also feature distinct vibronic progressions associated with the stretching vibrations of the B–N bonds. In contrast, TADF was observed for 125 possessing Δ*E*_ST_ of 0.19 eV. Enantiomers of this double helicene, consisting of [6]- and [7]helicene moieties, were successfully resolved by HPLC, showing mirror-image CPL with |*g*_lum_| reaching 1.41 × 10^−3^ in toluene and 1.36 × 10^−3^ in doped film.^134^

As shown by Zhang and Duan, fusion of a MR-fragment with the conventional rigid PAHs can lead to suppression of vibronic progressions present in the spectra of these PAHs.^160^ MR-TADF emitters 135 and 136 (Fig. 5), comprising an indolocarbazole-type rigid core, revealed narrowband emission with FWHM of 21–22 nm (0.09 eV) and *Φ*_PL_ of close to unity. The shoulder peak intensities of 135 and 136 were reduced compared to the parent indolocarbazole. Their *λ*_em_ were positioned at *ca.* 520, which – together with small FWHM – gave rise to the ultrapure green MR-TADF emitters.

Two other green emitters, 137 ^161^ and 126,^135^ (Fig. 5) were also prepared through the addition of the respective PAH fragments, *i.e.* phenanthrene and pyrene, respectively, to the original MR-TADF core. These two compounds were synthesized by post-functionalization of the boron-containing core, leading to the construction of a [6]helicene framework in each case. As opposed to 135 and 136, boron atoms in 137 and 126 are placed on the outer helicene rim. Both helicenes show intense green fluorescence emission with *Φ*_PL_ of 0.96 and 0.90 in degassed toluene and the maxima located at 523 and 527 nm, respectively. The corresponding FWHM of 34 and 35 nm were narrow and allowed to obtain pure green emission. Their Δ*E*_ST_ values (0.14 and 0.20 eV) were sufficiently low to facilitate the TADF process. However, the *g*_lum_ values determined for the doped films of (*P*)- and (*M*)-126 (3 wt%) were low: −2.74/+3.67 × 10^−4^.

Another green emitter, 138 (Fig. 5), with an excellent *Φ*_PL_ of 0.98 in doped film was constructed from two [5]helicene units with an overlapping benzoid ring.^162^ This double helicene, consisting of two 1,4-thiazine rings, shows *λ*_em_ at 520 nm, FWHM of 46 nm and Δ*E*_ST_ of 0.18 in toluene. Due to the presence of sulphur atoms, the contribution of delayed fluorescence prevailed over the prompt component (69 *vs.* 29, respectively, for doped film). The |*g*_lum_| in toluene reached a decent value of 2.1 × 10^−3^, while in doped films, the dissymmetry factors were determined as +1.3 × 10^−3^ for (*P*)-138 and −2.0 × 10^−3^ for (*M*)-138, thus differed quite significantly for both enantiomers. In particular the latter value indicated no racemization taking place during thermal deposition.

Configurational stable 127–129 (see Fig. 4) consisting of only four angularly fused rings in a helical framework also benefited from the presence of a thiazine ring.^136^ The introduction of sulphur resulted in enhanced *k*_RISC_ on the order of 10^5^ s^−1^, small Δ*E*_ST_ of 0.16 for 127, and 0.14 for both 128 and 129. In addition, their high *Φ*_PL_ of 0.72, 0.88, and 0.87 in toluene contained a substantial part of a delayed component. The narrow bands of 128 and 129 (FWHM of 43 nm and 44 nm (*ca.* 0.21 eV)) with the maxima positioned at 500 and 497 nm correspond to the green emission color. The enantiomers of 128 and 129 could be resolved and characterized to show moderate |*g*_lum_| of 1.0 × 10^−3^ to 2.0 × 10^−3^.

This class of compounds exhibit, in general, excellent properties, such as strong absorption and emission, and high thermal stability. However, the |*g*_lum_| reported for MR-TADF materials are relatively low, far from the actual applications, and have tremendous room for improvement, which would require expanding the structural variety in the search for structure–property relationships.

The last class of compounds discussed herein are helically chiral PAHs consisting of only C and B. The data regarding their chiroptical properties are essentially, with one exception, not available yet, since derivatives published to date are fluxional at room temperature. Some of these non-planar B-doped compounds, however, possess attractive emissive and electronic properties. As described by Wagner, [4]helicene 87 exhibits green emission with a remarkable *Φ*_FL_ of 0.81 in cyclohexane and somewhat lower values in benzene and CHCl_3_ (0.75 and 0.63), although its emission is substantially quenched at higher concentrations.^90^ Hatakeyama reported *Φ*_FL_ of 0.90 in toluene for 87.^92^ This compound also shows weak solvatochromism (*λ*_em_ from 485 nm in cyclohexane to 500 nm in CHCl_3_).^90^ Surprisingly, the emission efficiency of configurationally stable 103 was essentially not reduced upon elongation. Its *λ*_em_ is centered at 587 nm and corresponds to the excellent *Φ*_FL_ of 0.77 in degassed CHCl_3_. On the other hand, the |*g*_lum_| value is average (1.4 × 10^−3^).^44^

Likewise, 93 with two aliphatic bridging units shows excellent emission efficiency of 0.79 in toluene and a red-shifted emission band (*λ*_em_ = 516 nm) due to the effective hyperconjugation.^92^ Upon enlargement of the system, the emission is slightly shifted to higher energies. Thus, *λ*_em_ in cyclohexane, benzene and CH_2_Cl_2_ of double [4]helicene 90 were positioned between 472 and 484 nm, while the *Φ*_FL_ in these solvents were also high (0.65–0.69). However, it is reduced in THF, which likely interacts with the Lewis acidic boron atoms.^91^ Lower *Φ*_FL_ were also recorded for helicenes, having B atoms on the outer helicene rim. *Φ*_FL_ of single helicenes 97a and 100, as well as double helicene 99 embedding a diborapyrene moiety are in the range of 0.19–0.24 in toluene.^94^ In double helicenes, fluorescence efficiency depends on the arrangement of boron atoms around the naphthalene moiety. *Φ*_FL_ of 101 with the boron atoms attached to the 1 and 6 positions is twice as high as that of 1,5-substituted naphthalene 99 (0.49 *vs.* 0.23 in toluene), although it is reduced again for triple helicene 102 (0.34). Upon extension of the chromophore, *λ*_em_ gradually red-shifts reaching 623 nm for the latter compound.^95^

Some of these boron-doped helically chiral compounds possess excellent optical properties. It is interesting though how the emission efficiency would change upon helical elongation of the system, necessary to achieve configurationally stable structures. It is reasonable to expect that the *Φ*_FL_ will be reduced, as in the case of other types of helicenes, although in a single case of configurationally stable B-doped helicene 103, a high *Φ*_FL_ has been retained.^44^ Another problem in some applications may be high Lewis acidity of these compounds and undesirable interactions with other components. As shown by Wagner,^91^ even the measurements of emission in (weakly) coordinating solvents can lead to a decrease or a complete quenching of their emission. This may be an obstacle in the fabrication of the optoelectronic devices. On the other hand, such interactions may be an asset for rationally designed systems.

## 4. Application of B-doped helicenes

The most prominent application of helically chiral boron compounds is as dopants in OLEDs. Due to their exceptional optical properties, they are commonly used as intrinsic CPL (I-CPL) emitters, where emission arises directly from chiral chromophores. On the other hand, studies of B-doped helicenes as semiconductors in transistors or batteries are exotic, with only single examples in each case.

### 4.1. Application as emitters in OLEDs

Excellent emissive properties of boron-containing compounds are a prerequisite for their application in the display industry. These features of achiral PAHs are frequently exploited in OLEDs. In contrast, the number of helically chiral derivatives with sufficient configurational stability is limited in this field. Below, we present several examples of boron helicenes that were applied as terminal emitters in emitting OLED layers with some key device characteristics, including external quantum efficiency (EQE), and CIE color coordinates.

Due to the structural requirements for achieving narrowband emission, such as multiple rings fused with the B-π-B and E-π-E (E = N, O, S) motifs in a *para* arrangement, the chromophores of helically chiral derivatives usually reach quite large sizes resulting in a green or red emission color. Some of these compounds, despite their sufficient or high configurational stability, were used in OLEDs as standard achiral emitters without exploiting their CPL properties in the devices under study. The blue-shifted photoluminescence can be achieved by reducing the electron-accepting nature of boron *via* introduction of a heteroatom–boron bond, or replacing more-electron rich nitrogen with oxygen. The second strategy is very effective, but the configurational stability of the resulting systems is compromised, resulting in derivatives that are fluxional at room temperature or their configurational stability is not sufficient for practical applications. Thus, blue (CP-)-OLEDs using MR emitters have yet to be realized.

It is also noteworthy that MR-TADF emitters, the major class of boron-containing helicenes studied in OLEDs, exhibit relatively low *k*_RISC_ leading to significant OLED efficiency roll-off. The longer residence time in T_1_ (long triplet exciton lifetimes) enhances the probability of other processes to occur in electroluminescent devices, such as singlet–triplet annihilation (STA), triplet–triplet annihilation (TTA) or triplet-polaron annihilation (TPA).^171^ To alleviate this problem, OLED devices are often fabricated using ternary systems as emissive layers (EMLs), consisting of a terminal emitter, a matrix, and an additional TADF photosensitizer (TADF-sensitized fluorescence (TSF)). The latter reveals better TADF characteristics than the MR-TADF emitter and is responsible for the triplet exciton energy transfer, *i.e.* upconversion of triplet excitons to singlet states of an additional dopant through efficient RISC. The subsequent long-range Förster energy transfer (FET) process leads to the excitation of a terminal MR-TADF emitter to the S_1_ state, from which radiative decay, that is fluorescence, takes place. Importantly, this approach can also be used for terminal emitters showing no TADF features.^172,173^ The host matrix must have higher S_1_ and T_1_ energy levels than those of the assistant dopants. Alternatively, phosphorescent emitters can be used as triplet sensitizers to harvest triplet excitons through energy transfer from the T_1_ state of a phosphorescent emitter to the S_1_ state of a terminal fluorescent emitter *via* FRET in the so-called phosphorescence-sensitized fluorescent OLEDs (PSF-OLEDs). The concentration of the additional dopant should be sufficiently high to ensure charge recombination on the photosensitizer rather than the terminal emitter. On the other hand, it should not exceed a certain value in order to avoid possible short-range Dexter energy transfer from the T_1_ state of the sensitizer to T_1_ of the terminal emitter or aggregation-caused quenching of luminescence.^172^

### 4.2. Red emitters

As revealed by thermogravimetric analysis (TGA), 40 shows excellent thermal stability with the decomposition temperature *T*_d_ (5% weigh-loss temperature) of 533 °C, indicating its suitability for thermal evaporation under high vacuum (<6 × 10^−5^ Pa) during OLED fabrication. Despite the rather low *k*_RISC_ of 40, the OLEDs were fabricated using the binary emitter layer composed of 40 as the emissive dopant (2 wt%) and 3,3′-di(9*H*-carbazol-9-yl)-1,1′-biphenyl (mCBP) as the host, leading to the significant efficiency roll-off. The resulting devices manifested narrowband red electroluminescence (EL) with a peak at 616 nm and the corresponding CIE chromaticity coordinates of (0.67, 0.33), FWHM value of 26 nm, and high EQE_max_ of 22.0%. The good performance is attributed not only to the high *Φ*_PL_ (0.79 in doped film) of these helicenes, but also to the horizontal dipole orientation, which increases the light outcoupling efficiency of devices.^68^

Due to the weak TADF nature of 41a and 41b, the OLEDs were fabricated using ternary emitter layer. The EML consisted of 3 wt% 41a/41b doped in the 4,4′-di(9*H*-carbazol-9-yl)-1,1′-biphenyl (CBP) matrix, and 30 wt% (bis(4-methyl-2-(3,5-dimethylphenyl)quinoline))Ir(iii) (tetramethylheptadionate) (Ir(mphmq)_2_tmd)^174^ as the phosphorescent sensitizer to improve the exciton harvesting under electrical excitation. High *Φ*_PL_ could be retained in the doped films due to the MR effect and the rigid geometries of 41a and 41b. Their PSF-OLEDs manifested EL emissions with the maxima at 664 nm and 686 nm, narrow FWHMs of 48 and 49 nm, and high EQE_max_ of 28.1% and 27.6%, respectively. The devices showed reasonable operational stability with LT_90_ (time of luminance decay to 90% of the initial value) of 125 and 151 h at an initial luminescence (*L*_0_) of 2000 cd m^−2^, respectively. However, their emission (CIE coordinates of (0.719, 0.280) and (0.721, 0.278) for 41a and 41b, respectively) falls in the deep red region, which deviates from the pure red color requirements given by BT.2020 standard. Interestingly, the devices with a binary EML showed only slightly lower EQEs, but suffered from a substantial efficiency roll-off.^70^

The hypsochromic shift of emission can be obtained by the introduction of B–N covalent bonds increasing the electron density on boron. This strategy allowed to shift *λ*_EL_ to 617 nm for CP-OLEDs of 125. The EML was composed of the exciplex-forming host, 9-(9,9′-spirobi[fluoren]-3-yl)-9′-phenyl-9*H*,9′*H*-3,3′-bicarbazole (SFBCz)/2-(3′-(9,9′-spirobi[fluoren]-3-yl)-[1,1′-biphenyl]-3-yl)-4,6-diphenyl-1,3,5-triazine (SFTRZ)^175^ (1 : 1), 1 wt% 125, and 15 wt% Ir(mphmq)_2_tmd sensitizer. The PSF-devices based on its (*M*,*M*)- and (*P*,*P*)-enantiomers manifested distinct CP-EL signals with good dissymmetry factors of up to +1.91 and −1.77 × 10^−3^, respectively, FWHM of 48 nm (0.15 eV), high EQE_max_ of over 34%, and a high ratio of horizontal dipole orientation. Importantly, the CIE coordinates of (0.667, 0.332) closely match the red color CIE coordinates of the widely accepted National Television System Committee (NTSC) standard (0.66, 0.33) and approach the requirements given by BT.2020 for ultra-high-resolution displays. Moreover, both enantiomers exhibit an extremely long LT_95_ of *ca.* 400 h at *L*_0_ of 10 000 cd m^−2^, indicating the exceptional operational stability of the 125-based devices.^134^

The *λ*_EL_ was even more hypsochromically shifted for OLEDs of 133 (525 nm) 134 (552 nm). Because 133 and 134 did not show TADF, the corresponding devices with a binary EML showed poor performance. The strategy selected to overcome the inefficient triplet harvesting in this case, was utilization of a ternary EML with as the TADF assistant dopant. The TSF-OLEDs were fabricated using 9-(3-(9*H*-carbazol-9-yl)phenyl)-9*H*-3,9′-bicarbazole (mCPBC), a wide-bandgap host with high-lying T_1_, 1 wt% terminal emitter and 30 wt% either 3CTF^176^ or DACT-II^177^ as the TADF sensitizers for 133 and 134, respectively.^159^ The devices showed either green (133) or yellow-green (134) electroluminescence with good overall performance, including high EQEmax of 27.6% and 24.6%, respectively, FWHM of 31 nm (0.13–0.14 eV), small efficiency roll-off, and good operational stability. Such high EQE values are due to their high ratios of horizontal dipole orientations and high *Φ*_PL_ values. However, the CIE coordinates of (0.306, 0.648) and (0.414, 0.571) for 133 and 134 do not comply with the BT.2020 standard.

Like in 133, the weaker short-range charge transfer can be achieved by replacing the nitrogen with an oxygen atom. Such a one-fold exchange in a PAH frameworks of 139 and 140 (Fig. 5) resulted in the red emitters containing one [7]- and one [5]helicene moiety.^178^ When embedded in a host matrix (1 wt%) together with a sensitizer, they produced EL devices with *λ*_EL_ of 632 and 645 nm, corresponding to the CIE coordinates of (0.689, 0.311) and (0.701, 0.301), and excellent EQE_max_ of 33.1 and 34.7%. Remarkably, the sensitized OLEDs constructed with 2 wt% 140 achieved the optimal coordinates (0.708, 0.292) according to the BT.2020 standard, although its EQE decreased with the increase of the 140 concertation in EML.

### 4.3. Green emitters

The green emitters possess smaller chromophores, usually with one helicene moiety. As in the case of red emitters, they have been mainly applied in conventional OLEDs, while the reports of their use in CP-OLEDs are rare. Accordingly, 135 and 136 (3 wt%) were doped in the mCBP host, together with the 3CTF (30 wt%) TADF dopant to construct TSF-OLEDs.^160^ The devices showed good EL performance (*λ*_EL_ = 523 nm, FWHM = 23 nm (0.09 eV), and EQE_max_ of 29.8–30.5%), green color with a favorable CIE *y* coordinate of 0.73–0.74, but with non-negligible roll-off. Even higher color purity was achieved for the top-emitting OLED using 135 (3 wt%) doped in mCBP, and an additional phosphorescent sensitizer, Ir(ppy)_2_acac. Notably, this OLED exhibited a high current efficiency of 220 cd A^−1^, reduced FWHM of 19 nm and CIE of (0.17, 0.78), essentially meeting the BT.2020 requirements for green color.

The OLED employing 137 as the emitter (3 wt%) in the 9-(2-(9-phenyl-9*H*-carbazol-3-yl)phenyl)-9*H*-3,9′-bicarbazole (PhCzBCz) host exhibits green emission with a peak at 528 nm, FWHM of 36 nm, and EQ_Emax_ of 35.1%. Its CIE coordinates of (0.26, 0.70) deviate somewhat from the optimal coordinates of the new color gamut standard.^161^

The enantiomers of structurally similar 126 (3 wt%) were applied in vacuum-deposited CP-OLEDs with the (9-(3-(6-phenyl-9*H*-pyrido[2,3-*b*]indol-9-yl)phenyl)-9*H*-3,9′-bicarbazole) (PhCbBCz) host to achieve EQE_max_ of *ca.* 30%. Here, the configurational stability of the emitter is critical to the operation of the devices. Owing to its high Δ*G*^‡^, essentially no racemization was observed during the thermal evaporation process (150 °C, <5 × 10^−4^ Pa). The ee values of the remaining enantiomer samples after repetitive evaporation and those of the initial samples were nearly identical. However, the devices show low *g*_EL_ of −4.37 × 10^−4^ and +4.35 × 10^−4^ for (*P*)-126 and (*M*)-126, respectively and the green emission with the sharp peaks at 532 nm and FWHMs of 37 nm, corresponding to CIE coordinates of (0.29, 0.68) that deviate from the BT.2020 standard.^135^

Higher |*g*_EL_| of 2.2 × 10^−3^ for and 1.2 × 10^−3^ were obtained for the OLEDs based (*M*)-138 and (*P*)-138, fabricated by thermal deposition. The devices with a binary EML, using (1,3-dihydro-1,1-dimethyl-3-(3-(4,6-diphenyl-1,3,5-triazin-2-yl)phenyl)indeno-[2,1-*b*]carbazole) (DMIC-TRZ) as host, exhibited green emission with *λ*_EL_ of 523–524 nm, FWHM of ≤50 nm and EQE_max_ of around 31%. Here, the host also served as a TADF sensitizer to ensure efficient energy transfer.^179^ However, the CIE coordinates of (0.26, 0.66) also deviate from the BT.2020 standard.^162^

The configurational stability of 128 and 129 is relatively low. Therefore, the corresponding CP-OLEDs were fabricated by solution processing. The binary EML was composed of an enantiomer of the MR-TADF emitter (3 wt% 128 and 1 wt% 129) doped in 9-(3-(9*H*-carbazol-9-yl)phenyl)-9*H*-carbazole-3-carbonitrile (mCPCN) to show green emission, FWHM of 0.23 eV (48–49 nm) and EQE_max_ of *ca.* 20% for 128 and up to 26.5% for 129, lower than for other chiral green emitters. On the other hand, the devices achieved good *g*_EL_ of +3.7 × 10^−3^/−3.1 × 10^−3^ and +1.9 × 10^−3^/−1.6 × 10^−3^ for (+)-/(−)-128 and (+)-/(−)-129, respectively. Additionally, the corresponding CIE coordinates of (0.186, 0.632)/(0.206, 0.635) and (0.173, 0.590)/(0.167, 0.603) approach the green color requirements of BT.2020. More specifically, the *x*-coordinate closely matches the optimal value. A major shortcoming of these devices is attributed to the significant efficiency roll-off due to the relatively slow RISC, similar to other MR-TADF emitters discussed in this article.^136^

Undoubtedly, these materials hold great potential for use in CP-OLEDs. The high EQE of these devices can be correlated with the high *Φ*_PL_ of the chiral MR-(TADF) emitters with rigid molecular frameworks and their dominant favorable horizontal emitting dipole orientation ratios. However, chiral blue emission has not yet been realized through the assembly of common MR building blocks. In addition, the devices experience a significant efficiency roll-off, especially when using a binary EML without any additional dopant. This is due to low *k*_RISC_, which increases the likelihood of undesired events to occur in the excited state. This is why either TADF or phosphorescent sensitizers are often added to EML to enhance triplet exciton harvesting and improve the overall device performance. Developing a binary emissive layer without any additional sensitizer would simplify device fabrication but would require more efficient TADF emitters.

### 4.4. Application as chiral inducers in CP-OLEDs

Another strategy to prepare an EML emitting CPL is based on the extrinsic CPL system (E-CPL). In this case, a helicene is used as a chiral inducer in combination with an emissive polymeric material to generate a chiral supramolecular phase. In the E-CPL strategy, the observed CPL originates from a supramolecular chiral phase rather than from a CPL emission of a helicene. Our group in collaboration with the Meerholz group has implemented one of the azaborole helicenes in CP-OLEDS. For these studies, we have selected azabora[7]helicene 13a due to its high configurational stability and ease of preparation of large quantities of optically pure samples.^57^ Since 13a showed CPL emission, we used it initially as a chiral emitter (I-CPL). However, its *Φ*_PL_ in the spin-coated film was only 0.08, an even lower *Φ*_PL_ were recorded for the doped films (5–15 wt%) in poly(9-vinylcarbazole) (PVK). This, combined with its relatively low|*g*_lum_|, did not allow to fabricate good devices. Therefore, we decided to use 13a as a chiral inducer (10 wt%) in combination with a green light emitting polymer, poly(dioctylfluorene-*co*-benzothiadiazole) (F8BT), in CP-OLEDs due to its favorable thermal stability. The blend was annealed at 180 °C for 15 min, above the glass transition of the polymer (*ca.* 100 °C). This step is essential for the formation of the chiral phase in the E-CPL strategy and requires high configurational stability of the chiral dopant. The fabricated devices manifested maximum *g*_EL_ of +0.54 and −0.49 for the (*M*)- and (*P*)-enantiomers, respectively, and good device performance. The difference in the absolute values is attributed to the different EML thickness. The E-CPL emission was first demonstrated in 2013 by Campbell and Fuchter for a blend of 1-aza[6]helicene and F8BT producing CP-OLEDs with |*g*_EL_| of 0.2.^180^ Meanwhile, some devices fabricated based on this concept have achieved the values of *ca.* unity.^181,182^ This shows a clear advantage of using a chiral dopant and an emissive polymer over conventional CP-OLEDs with chiral emitters. The downside of these devices is however broad emission, which has a detrimental effect on color purity.

### 4.5. Application as semiconductors

The electrophilic nature of boron atoms renders boron-doped aromatic compounds attractive candidates for semiconducting materials. Incorporation of boron, often in combination with other heteroatoms, *e.g.* nitrogen, can provide materials with low-lying LUMO levels, high charge carrier mobilities and good photovoltaic performance, as demonstrated for various four- and three-coordinate boron compounds.^45–47,183,184^ In addition, the presence of a three-coordinated boron can facilitate the solution processing. As shown by Yamaguchi, the poor solubility of the material can be overcome through the reversible formation of the boron–pyridine adduct that disrupts its close packing in the solid state. The ligand can be eventually removed during the annealing process.^185^

The use of configurationally stable helically chiral derivatives is however scarce in this field. The enantiomers of O–B–O-based double [5]helicene 46a formed a herringbone-type packing arrangement in the solid state, showing potential for use as a semiconductive material. The film of *rac*-46a was prepared by vacuum deposition, which would not be appropriate for the preparation of films from enantioenriched samples due to the low Δ*G*^‡^ (see Section 3.1). The film exhibited balanced ambipolar conductivity with hole mobility (*μ*_h_) of 5.7 × 10^−3^ cm^2^ V^−1^ s^−1^ and electron mobility (*μ*_e_) of 7.9 × 10^−3^ cm^2^ V^−1^ s^−1^, as determined by time-of-flight (TOF) measurements, which are commonly used to measure charge carrier mobility of materials for OLEDs.^71^ Notably, azaboradibenzo[6]helicene 59 showed unprecedented carrier inversion between the racemate and the single enantiomer. While p-type semiconductivity (*μ*_h_ = 4.6 × 10^−4^ cm^2^ V^−1^ s^−1^) was observed for the racemate, the film of the enantiomer exhibited preferably n-type semiconductivity (*μ*_e_ = 4.5 × 10^−3^ cm^2^ V^−1^ s^−1^*vs. μ*_h_ = 7.9 × 10^−4^ cm^2^ V^−1^ s^−1^). These differences originate from differences in the packing arrangements of the racemate and the enantiomer.^77^ Even though these mobilities are relatively low, the study is highly interesting as it shows the impact of chirality on charge carrier mobility. This has also been observed for other materials, but such studies are extremely rare.

### 4.6. Application in batteries

Currently, organic compounds have been intensively investigated as electrode materials for batteries. They offer numerous advantages over inorganic materials, such as flexibility, structural diversity, high power and energy density, and sustainability, as they do not require the use of transition metals.^186,187^ However, these materials also have significant shortcomings, including low electronic conductivity and dissolution of the organic material in the electrolyte. The latter is one of the major factors contributing to poor cycling performance of the organic material electrodes.^188,189^ This problem can be tackled by the integration of the redox active moiety into polymeric materials.^190,191^

The use of boron-containing π-conjugated compounds in batteries is extremely rare. Even more unique in this field are helically chiral derivatives. In 2019, Yoshikawa and Hatakeyama reported the first example of application of boron-doped double [5]helicene 52b as a cathode material in a lithium-ion battery.^75^ This four-coordinate boron compound showed two reversible reduction processes at −0.97 and −1.64 V and thereby low-lying LUMO level, which was attractive for its use in lithium-ion batteries (LIBs). The fabricated LIB exhibited rather poor performance compared to the state of the art organic cathode materials.^186^ The second discharge capacity of 93 mA h g^−1^ was gradually reduced to reach the value of *ca.* 63 mA h g^−1^ after only 20 cycles, while high coulombic efficiency of over 90% was maintained. As this is the only report on the use of boron-containing helicenes in batteries, further studies are needed to assess the potential of this class of molecules and the corresponding polymeric materials for applications in energy-storage devices.

## 5. Conclusion & future perspectives

In this perspective article, we have summarized the major achievements in the new and rapidly developing field of boron-containing helicenes, discussed the challenges in the synthesis and design of materials with desired properties, and pointed out the area of this research that should be further developed in the future.

In only a decade, remarkable progress has been made in the synthesis of these highly interesting compounds. The vast majority of the synthetic approaches toward boron-doped helicenes rely on the introduction of boron atom(s) in the final step. The concomitant boracycle ring(s) formation proceeds against steric strain present in both the precursors and the target helicenes, rendering the synthesis of these compounds more challenging. In addition, sterically demanding precursors, whose synthesis is not covered in this article, can pose significant difficulties, and even represent a bottleneck in the entire synthetic sequence. Therefore, it is highly important to develop new robust synthetic protocols that would facilitate these demanding transformations. More general, finding new methods and approaches that would circumvent the limitations of the existing and traditional ones would accelerate the progress in the field of chiral boron functional materials.

Another challenge is to provide easy access to optically pure or enriched compounds. Currently, the majority of boron helicenes have been prepared as racemates and subsequently resolved by HPLC on a chiral stationary phase. This is definitely a major factor hindering the development of chiral boron-containing functional materials. The need for the chromatographic separation restricts the access to optically pure or enriched samples in amounts that are required for testing a given compound in devices. In addition, finding suitable conditions for chiral resolution can be tedious, extremely difficult, and sometimes even unsuccessful. These problems could be solved by developing efficient protocols for the stereoselective synthesis of different subclasses of boron helicenes, which would be applicable to a broad substrate scope. Except for a few examples of stereoselective synthesis of boron helicenes, including our stereospecific synthesis of laterally extended azaboroles *via* axial-to-helical chirality transfer, which is, on the other hand, limited by the access to optically active biaryls,^58^ this area is yet to be explored.

Nonetheless, advances in the synthesis have made it possible to access a wide range of boron helicenes, to study their properties, and to establish certain structure–property relationships. In this Perspective article, we focused on emissive properties, as the major field of application of these compounds are (CP-)OLEDs. The majority of boron-containing helicenes exhibits appreciable photoluminescence quantum yields, which can be enhanced by the extension of the chromophore. Therefore, efficient boron emitters emit typically green to red light, while configurationally stable blue emitters, suitable for applications in OLEDs are yet to be developed.

One of the subclasses of helicenes with exceptionally strong emission are MR-TADF emitters, many of which have been used in OLEDs. The general problem to overcome is their rather low *k*_RISC_ due to the relatively high Δ*E*_ST_, which contributes to the device efficiency roll-off. Currently, ternary EMLs with an additional sensitizer are used to improve exciton harvesting. Accelerating the RISC would allow the use of a binary layer and thus simplify device fabrication. Moreover, only a few of these helicenes have been tested in CP-OLEDs, typically achieving an average *g*_EL_, which correlates with the material property *g*_PL_.

In general, the strategy for improving the dissymmetry factors, should focus on engineering the magnetic dipole moment so that S_1_ → S_0_ is magnetically allowed,^150^ and optimizing angle *θ*_*μ*,*m*_ between the *m* and *μ* vectors. Indeed, some correlations have been observed, including the relation between the molecular symmetry and *g*_lum_,^149,157,192^ as well as the area of the inner cavity of the helix and *m*.^193^ However, despite considerable efforts to elucidate the design principle of CPL materials, a comprehensive understanding has not yet been achieved. Therefore, the development of superior CPL materials requires systematic structural modification of molecular scaffolds that have already been identified as demonstrating satisfactory *g*_lum_ values and decent photoluminescence quantum yields. While this is a promising approach, new scaffolds should also be investigated. It appears that the progress in this field will be mainly achieved by combined efforts of many groups, working on different types of boron-containing helicenes and other chiral π-conjugated compounds, optimizing their molecular structures in a trial-and-error manner.

These studies should also consider other essential factors, such as chemical stability, photostability, resistance to racemization and color purity for CP emitters, as these parameters, except for the configurational stability, cannot be easily predicted. Obtaining materials that excel in each of these areas would be highly desirable. However, it has been demonstrated by numerous studies that the tested compounds typically fall short in at least one (more often in several) of these parameters. For instance, a compound may display appreciable *g*_lum_ and angle *θ*_*μ*,*m*_ but relatively low configurational stability, which limits the available processing methods, excludes the possibility of annealing and reduces the device lifetime due to possible racemization. On the other hand, the compound displaying good optical properties and high enantiomerization or diastereomerization barrier may undergo slow degradation upon exposure to light. Thus, it is highly challenging to meet all the requirements for a certain application in a single molecule or material.

In addition, to realize real-world devices, the multivariate optimization of chiral materials has to be complemented by the general progress in device physics and fabrication, increasing the complexity of this nontrivial task.

By targeting these aspects, boron helicenes have a chance to move from fundamental academic research to practical application in CP-OLED devices in the foreseeable future. Apart from the use of B-helicenes in OLEDs, they are highly interesting for application in bioimaging and as semiconducting materials or chiral dopants, although such studies are still quite exotic. We expect that the increasing activity in the field of boron-containing helicenes will lead to breakthroughs in these and other areas beyond the use of these compounds as emitters.

## Author contributions

ANK conceptualized, ANK, PTG, and KRN wrote, ANK edited, ANK and PTG reviewed the Perspective.

## Conflicts of interest

There are no conflicts to declare.

## Supplementary Material
